# Deciphering the origin of giant magnetic anisotropy and fast quantum tunnelling in Rhenium(IV) single-molecule magnets

**DOI:** 10.1038/ncomms10669

**Published:** 2016-02-17

**Authors:** Saurabh Kumar Singh, Gopalan Rajaraman

**Affiliations:** 1 Department of Chemistry, Indian Institute of Technology, Bombay Powai, Mumbai 400076, India

## Abstract

Single-molecule magnets represent a promising route to achieve potential applications such as high-density information storage and spintronics devices. Among others, 4*d*/5*d* elements such as Re(IV) ion are found to exhibit very large magnetic anisotropy, and inclusion of this ion-aggregated clusters yields several attractive molecular magnets. Here, using *ab intio* calculations, we unravel the source of giant magnetic anisotropy associated with the Re(IV) ions by studying a series of mononuclear Re(IV) six coordinate complexes. The low-lying doublet states are found to be responsible for large magnetic anisotropy and the sign of the axial zero-field splitting parameter (*D*) can be categorically predicted based on the position of the ligand coordination. Large transverse anisotropy along with large hyperfine interactions opens up multiple relaxation channels leading to a fast quantum tunnelling of the magnetization (QTM) process. Enhancing the Re-ligand covalency is found to significantly quench the QTM process.

## Abstract (web summary)

Rhenium(IV) complexes are magnetically anisotropic although the origin of this anisotropy is poorly explored compared to 3*d* transition metals and lanthanides. Here, the authors computationally examine the effects of ligand donor ability and structural distortion on magnetic anisotropy for a series of rhenium(IV) complexes.

## Graphical abstract

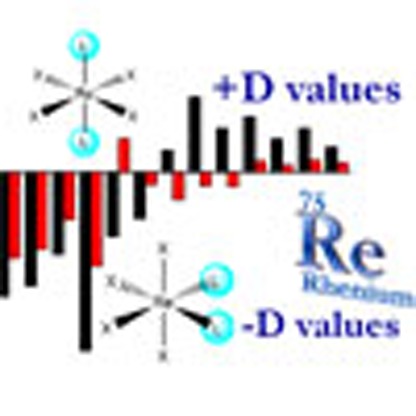

In the quest of single-molecule magnets (SMMs)^1^,^2^,^3^,^4^,^5^,^6^ with enhanced magnetic properties, magnetic anisotropy is found to be the most influential parameter, which governs the barrier height for slow relaxation of magnetization^7^,^8^,^9^,^10^,^11^. Owing to inherently large magnetic anisotropy, lanthanide-based complexes are promising candidate for single-ion magnets^2^,^3^,^5^,^12^,^13^,^14^,^15^,^16^,^17^ and mononuclear SMMs based on transition metal ions are relatively scarce in the literature, as stronger ligand field interactions suppress the orbital contributions to the anisotropy and hence the barrier heights (*U*_eff_)^18^,^19^,^20^,^21^,^22^,^23^,^24^,^25^. In the past few years, late-transition metal ions have gained much attention in the area of SMMs. The diffused magnetic orbitals of the 4*d*/5*d* ions translate stronger magnetic exchange, whereas larger spin-orbit coupling constants (SOCs) exhibited by these ions^26^, often lead to highly anisotropic ground state (highly anisotropic *g*-tensors with an unusually large zero-field splitting values (ZFS)). These two essential conditions along with a possibility of exhibiting anisotropic/anti-symmetric exchange makes this class of molecules ideal for observing SMM behaviour^27^,^28^,^29^,^30^,^31^,^32^,^33^. Owing to these advantages, these 4*d*/5*d* ions show better SMM behaviour compared with their 3*d* congeners at many occasions^27^,^28^,^31^,^32^,^33^. Among the 4*d*/5*d* ions, the chemistry of Re(IV) metal ion is very rich as they have been successfully used to isolate several single-chain magnets (SCMs)/SMMs with an attractive *U*_eff_ values^26^,^34^,^35^,^36^,^37^,^38^,^39^,^40^,^41^. Apart from rich magnetic studies, these Re(IV) complexes are also explored in the development of the new anticancer drugs^42^.

Despite several years of comprehensive experimental efforts in designing Re(IV) ion-based SMMs/SCMs, the origin of giant magnetic anisotropy is not well understood^39^. As in most of the cases, the axial ZFS (*D*) values are extracted using magnetization measurements, which are known to be insensitive to the sign and strength of the *D* parameter^41^,^43^,^44^,^45^,^46^,^47^,^48^,^49^,^50^,^51^. On other hand, the most promising high frequency-electron paramagnetic resonance (HF-EPR) technique has its own limitation, where the sign can be accurately determined but such large magnitude of *D* are often difficult to estimate^39^. An alternate solution to resolve the ambiguities in the sign/magnitude of *D* value is to analyse the ZFS parameter using *ab initio* calculations^9^,^22^,^39^,^52^,^53^,^54^,^55^,^56^,^57^, which has been widely used in this respect. Moreover, strategic designing of new generation SMMs based on Re(IV) ions requires a thorough understanding of the nature ZFS and how the magnitude and the sign of the *D* and *E* vary depending on the ligand field environment. As the magnitude of *E* and the hyperfine interactions are correlated to the quantum tunnelling of magnetization (QTM)^58^, the possibility to fine tuning these values is of paramount importance in this area.

The goal of the present communication is to gain a thorough understanding of the magnetic anisotropy in six coordinate Re(IV) complexes using state-of-the-art *ab initio* calculations. By modelling structurally diverse 13 mononuclear six coordinate Re(IV) complexes^39^,^41^,^43^,^44^,^45^,^46^,^47^,^48^,^49^,^50^,^51^, we aim to answer the following intriguing questions (i) What is the suitable theoretical methodology to compute ZFS parameters in 5*d* transition metal ions such as Re(IV) complexes? (ii) What is the origin of giant *D* values and is there a correlation between the nature of the donor atoms and the sign of the *D* values in these complexes? (iii) What is mechanism of magnetic relaxation in Re(IV) single-ion magnets and how this is influenced by the metal–ligand covalency?

## Results

### Magnetic anisotropy and spin-Hamiltonian

The free Re(IV) ion is a *d*^3^ Kramers ion with a ^4^F ground state term, which splits into three states ^4^A_2g_, ^4^T_2g_ and ^4^T_1g_ with the ^4^A_2g_ being the ground state in an octahedral environment. Due to perfect cubic symmetry, pure octahedral complexes do not possesses any ZFS, however, any distortions from the octahedral geometry are expected to yield large *D* values via mixing of the subsequent excited states because of very large SOC (*λ*∼1,000 cm^−1^). To begin with, we have studied the homoleptic [ReCl_6_]^2−^ model complex in tetragonal environment to analyse the origin of ZFS. Abragam and Bleaney have proposed a qualitative equation to predict the sign of the *D* values of a tetragonally distorted *d*^3^ ion.


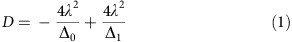


where *λ* is the SOC and Δ_0_, Δ_1_ is the tetragonal distortion parameter. If Δ_0_<Δ_1_, the sign is predicted to be negative, whereas for Δ_0_>Δ_1_, the sign is predicted to be positive. To corroborate this qualitative analysis, we have performed *ab initio* calculations, using complete active self consistent field (CASSCF) and CASPT2 methods incorporating spin-orbit effects with the RASSI-SO module in MOLCAS^59^,^60^. A positive *D* value of +19.3 cm^−1^ has been obtained for axially elongated model, whereas a negative *D* value of −24.3 cm^−1^ has been observed for axially compressed D_4h_ [ReCl_6_]^2−^ model complex (see Supplementary Fig. 1 and Supplementary Table 1 for details). The energy splitting pattern of the first three same spin-free states (^4^T_2g_(F)) are arranged as expected based on the ligand field theory and the sign of the *D* values computed using CASPT2 are in line with the expected values based on the equation (1). Although CASSCF calculations predict a similar splitting pattern of first three same spin-free states, it fails to reproduce the correct sign of the *D* values compared with CASPT2 methods for both elongated and compressed geometries. This suggests that spin-flip states rather than same spin-free states govern the sign as well as magnitude of *D* values for 5*d* elements such as Re(IV) ion. Hence, here after all the results discussed are performed at CASPT2 level of theory (vide infra).

**Figure 1:**
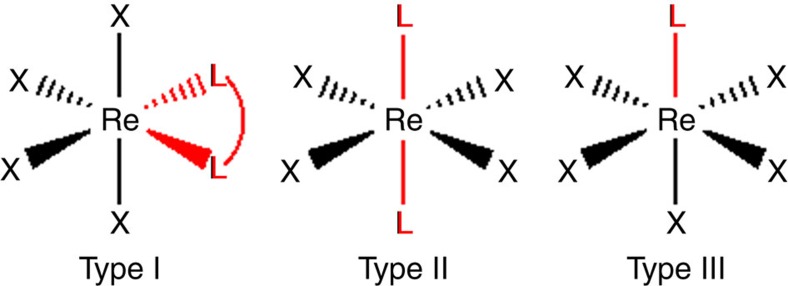
Structural topology. Classifications of substituted hexa halo Re(IV) complexes (where X=Cl, Br and L=coordinating ligand).

**Figure 2:**
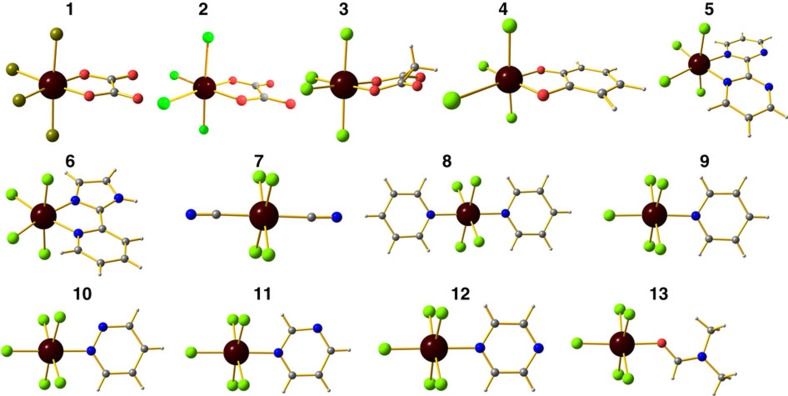
X-ray crystal structures. Crystal structure of Re(IV) mononuclear complexes. Colour code: dark brown, Re; pale brown, Br; light green, Cl; blue, N; grey, C; white, H. [ReBr_4_(ox)]^2–^ (**1**); [ReCl_4_(ox)]^2–^ (**2**); [ReCl_4_(mal)]^2–^ (**3**); [ReCl_4_(cat)]^2–^ (**4**); [ReCl_4_(bpym)] (**5**); [ReCl_4_(pyim)] (**6**); [ReCl_4_(CN)_2_]^2–^ (**7**); [ReCl_4_(py)_2_] (**8**); [ReCl_4_(py)]^–^ (**9**); [ReCl_5_(pyz)]^–^ (**10**); [ReCl_5_(pyd)]^–^ (**11**); [ReCl_5_(pym)]^–^ (**12**); [ReCl_4_dmf]^−^ (**13**). bpym, 2,2′-bipyrimidine; cat, catechol; dmf, dimethyl formamide; mal, malonato; ox, oxalato; pyim, 2-(2′-pyridyl)imidazole; py, pyridine; pyd, pyridazine; pym, pyrimidine; pyz, pyrazine.

To further understand the nature of *D* and *E* values, we have selected 13 mononuclear Re(IV) complexes and classified into three categories type-I: [ReX_4_(L)] (where L=a bidentate ligand on the equatorial plane), type-II: [ReX_4_(L)_2_] (where L=monodentate ligand in the axial positions) and type-III [ReX_5_(L)]^–^ (where L=monodentate ligand; see Figs 1 and 2 for details). Continuous symmetry measure analysis (SHAPE)^61^ of the X-ray structures reveals that all the complexes are in the distorted octahedral geometry (see Supplementary Fig. 2 and Supplementary Tables 2 and 3 for details).

### Sign and magnitude of ZFS parameter for 1–13

Calculations reveal that eight spin-free states corresponding to ^2^G states are found to be low-lying and thus are expected to contribute significantly to the *D* values via spin-flip excitations in all complexes **1**–**13** studied (see Supplementary Figs 3 and 4, Supplementary Tables 4–16 and Supplementary Note 1 for details). The MS-CASPT2+RASSI-SO computed *D*, *E* and the first spin-free excitation energies for all complexes are depicted in Table 1. For complexes **1**–**6**, large negative *D* and significantly large |*E*/*D*| values, with *D* as high as ±132 cm^−1^ (for **4**) have been witnessed. On other hand, complexes of type II and type III categories (**7**–**13**) found to posses positive ZFS parameter with *D* as high as +55 cm^−1^ has been noticed (for **8**; see Supplementary Fig. 5 and for the orientation of *D* tensor). The magnetic susceptibility and powder magnetization data computed for **1**–**13** reproduces nicely the experimental behaviour, adding confidence to the computed values (see Supplementary Figs 6–10 and Supplementary Note 2 for details). Simulation of HF-EPR spectra reported earlier^35^,^39^ confirm the negative sign of the *D* with a large *E*/*D* values for complexes **1** and **2**, with the *D* estimated to be ca −73 and −57 cm^−1^, respectively. Calculations yield *D* value of −93 and −85 cm^−1^ for complexes **1** and **2**, where both the sign as well as the magnitude of the *D* values are correctly reproduced compared with the experimental values. More importantly, the magnitude of the |*E*/*D*| values and *g*-tensors (both pseudo-spin 1/2 and 3/2), which are precisely estimated from the experiments are very well reproduced in our calculations (see Supplementary Tables 17 and 18 for details).

For complex **6**, the magnetization data^41^ yield an estimate of *D* as −14.4 cm^−1^, which is in agreement with CASSCF results (−21 cm^−1^, see Supplementary Table 17) but in disagreement with MS-CASPT2 values (+16.2 cm^−1^, see Table 1). However, HF-EPR experiments performed lately^35^, where both the magnitude as well as the sign of the *D* value is estimated accurately, places the *D* value to be +11 cm^−1^. This highlights the issue of obtaining the sign/magnitude of the *D* value from the magnetization data and also emphasise the need for CASPT2 approach and hence incorporation of dynamic correlation to correctly reproduce the sign of the *D* values^62^,^63^. Inclusion of dynamic correlation on CASSCF computed wavefunctions drastically stabilizes the doublet states compared with the computed CASSCF states, leading to a pronounced contribution from these states to the *D* values as discussed earlier (see Supplementary Fig. 3 for details). Moreover, either pseudo-spin or effective Hamiltonian approach^62^ needs to be employed to extract the ZFS parameters as other theoretical methodologies found to yield ambiguous sign and magnitude of *D* values (see Supplementary Tables 19 and 20 for details).

**Figure 3:**
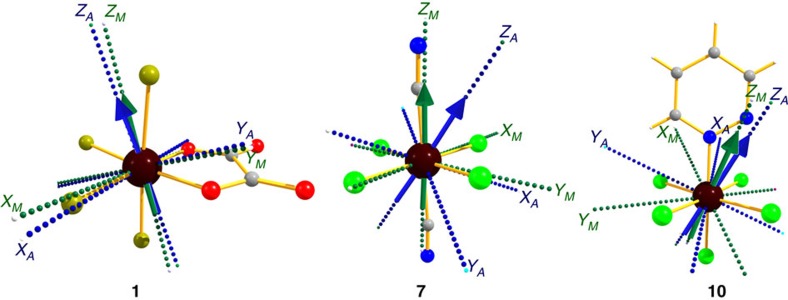
Orientation of *g* and *D* tensors. SINGLE_ANISO computed main magnetic (*X*_*M*_, *Y*_*M*_ and *Z*_*M*_) axes representing *g*-tensors orientation and main anisotropic axes (*X*_*A*_, *Y*_*A*_ and *Z*_*A*_) representing *D* tensors orientation for complexes **1**, **7** and **10**.

The SINGLE_ANISO computed orientations of the main anisotropic axes (*D*_*XX*_, *D*_*YY*_ and *D*_*ZZ*_) and main magnetic axes (*g*_*XX*_, *g*_*YY*_ and *g*_*ZZ*_) for all the complexes **1**–**13** are provided in Fig. 3 and Supplementary Fig. 5. It is evident from the figures that, the principal anisotropic axes (*D*_*ZZ*_) are oriented towards the L_ax_–R–L_ax_ (molecular –*z* axis) direction, however, a significant tilt from this axis is witnessed^64^. Moreover, the main magnetic axes (*g*_*XX*_, *g*_*YY*_ and *g*_*ZZ*_) and main anisotropic axes (*D*_*XX*_, *D*_*YY*_ and *D*_*ZZ*_) do not coincide with each other and such non-coincidence has been previously noticed by Askevold *et al*.^65^ The orientation of the *D*_*ZZ*_ axis is tilted by 28.8°, 36° and 33.7° from their molecular –*z* axis for complexes **1**, **7** and **10**, respectively. Large structural distortions and the associated large |*E*/*D*| values are responsible for such deviations. Larger |*E*/*D*| values detected in complex **7** has larger tilt compared with complex **1,** where smaller |*E*/*D*| value leads to smaller tilt. This trend is visible also for other structures. However, in the rhombic limit, the nature of the easy axis is ambiguous, as even the small structural distortions flip the Eigen values and hence the orientation of the easy axis of magnetization. This can be better visualized for complex **4**, where presence of large |*E*/*D*| value of 0.30 causes the flipping of *D*_*ZZ*_ axis to the equatorial plane.

### Origin of ZFS in complexes **1**–**13**

**Figure 4:**
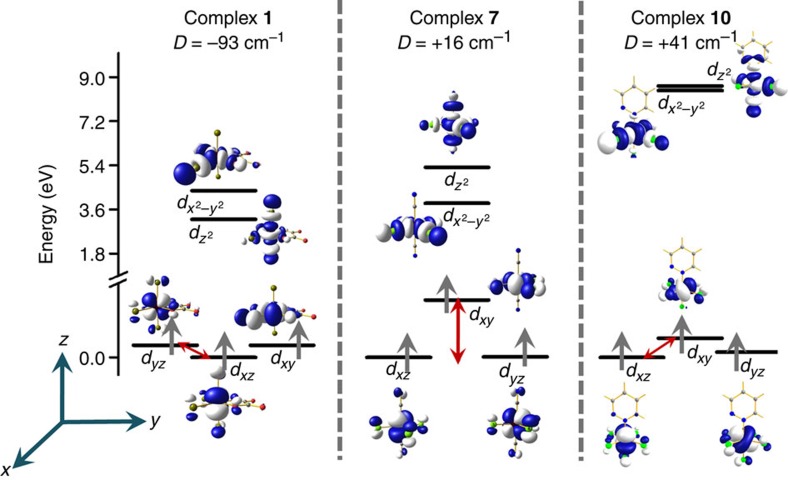
Molecular orbital analysis and nature of excitations. Computed *d*-orbital ordering for complexes **1**, **7** and **10**. The iso-density surface plotted with the iso-value of 0.02 e^−^/bohr^3^. The double headed arrow represents the gap between the orbitals, which are contributing significantly to the *D* value. The orbitals which are appeared as degenerate in the figure are not strictly degenerate due to symmetry arguments.

To shed light on the sign of ZFS, we have analysed the molecular orbitals (MOs) of complexes **1** and **7** and **10** as a representative examples for type I, II and III defined earlier (see Fig. 4 for details). For **1**, presence of unsymmetrical ligand in the equatorial position leads to the *d*_*xz*_ orbital being the lowest lying in energy followed by degenerate *d*_*yz*_ and *d*_*xy*_ orbitals. The π* orbitals of the oxygen donors interact rather strongly with the *d*_*yz*_ and *d*_*xy*_ orbitals because of acute ∠O-Re-O bite angle. The *d*_*xz*_ orbital on the other hand faces less repulsion leading to a slight stabilization. Besides, stronger σ* interaction by the oxalate ligand lead to destabilization of the *d*_*x*^2^__–*y*^2^_ orbital compared with the *d*_*z*^2^_ orbital. This orbital ordering has the following consequences to the *D* values: (i) all spin-conserved excitations from the *d*_*xy*_, *d*_*xz*_ and *d*_*yz*_ orbitals to the vacant *d*_*z*^2^_ orbital contribute to positive *D* values. This is affirmed by an additional calculation incorporating only the quartet states in the estimation of *D* and this yield a positive *D* value (+13 cm^−1^). (ii) Spin-flip excitations from the *d*_*xz*_ to the *d*_*yz*_ orbitals contribute to negative *D* values (excitation between same |m_l_| levels). As the gap between these two orbitals is very small (667 cm^−1^), this transition governs both the sign and the magnitude of the *D* value for this complex. A similar pattern predicted for complexes **2**–**6** rationalize the observed negative sign for these complexes.

Strong π acceptor cyanide ligand stabilizes the *d*_*xz*_ and *d*_*yz*_ orbitals via *p*π–*d*π interactions compared with the *d*_*xy*_ orbital. Stronger σ-donation along the axial directions destabilizes the *d*_*z*^2^_ orbital leading to different orbital arrangement compared with complex **1**. This orbital arrangement has the following consequences to the *D* value: (i) the spin-conserved *d*_*xy*_→*d*_*x*^2^__–*y*^2^_ transition contributes to the negative *D* value, affirmed by an additional calculation incorporating only the quartets in the estimation of *D* and this yields −26 cm^−1^ as the *D* value, (ii) the spin-flip excitations from the degenerate *d*_*xz*_–*d*_*yz*_ orbitals to the *d*_*xy*_ orbital contribute to the positive *D* value (the gap here is 3,123 cm^−1^). Here also the later term dominates leading to an overall positive *D* value for **7**.

For complex **10**, positive *D* value is expected as the splitting pattern is found to be very similar to that of complex **7** (Fig. 4). However, the presence of weak π-donor pyridazine ligand on the axial position reduces the splitting between the *d*_*xz*_–*d*_*yz*_ orbital and the *d*_*xy*_ orbital (1,044 cm^−1^). This suggests that much less energy is required to flip the spin in complex **10** compared with complex **7**, thus the *D* value is expected to be large in this case. A similar splitting pattern is predicted for complexes **8**, **9** and **11**–**13** rationalize the observed positive *D* values for these complexes.

### Rationale for the observed variation in the ZFS parameter

Among **1**–**6**, the equatorial positions are occupied by π-donor ligand, except in case of complex **5** where 2,2′-bipyrimidine (bpym) ligand serves as a weak π-acceptor ligand. Independent of the nature of the ligand (π-donor versus π-acceptor), in complexes **1**–**6**, the *d*_*yz*_→*d*_*xz*_ transition dominates the *D* value over other transitions, leading to a large negative *D* values. To understand the large difference in the *D* values of complexes **1** and **2**, we have performed additional calculations on model complexes where Cl in complex **1** is modelled as Br maintaining Re–Br distance same as that of complex **2**. For this model, the *D* is estimated to be −86 cm^−1^ compared with −85 cm^−1^ for the Cl analogue and this suggest that apart from the spin-orbit coupling of Br, the structural distortion such as –*cis* angles play an important role in determining the strength of the *D* value (see Supplementary Table 3 for selected structural parameters of complexes **1** and **2**)^55^.

**Figure 5:**
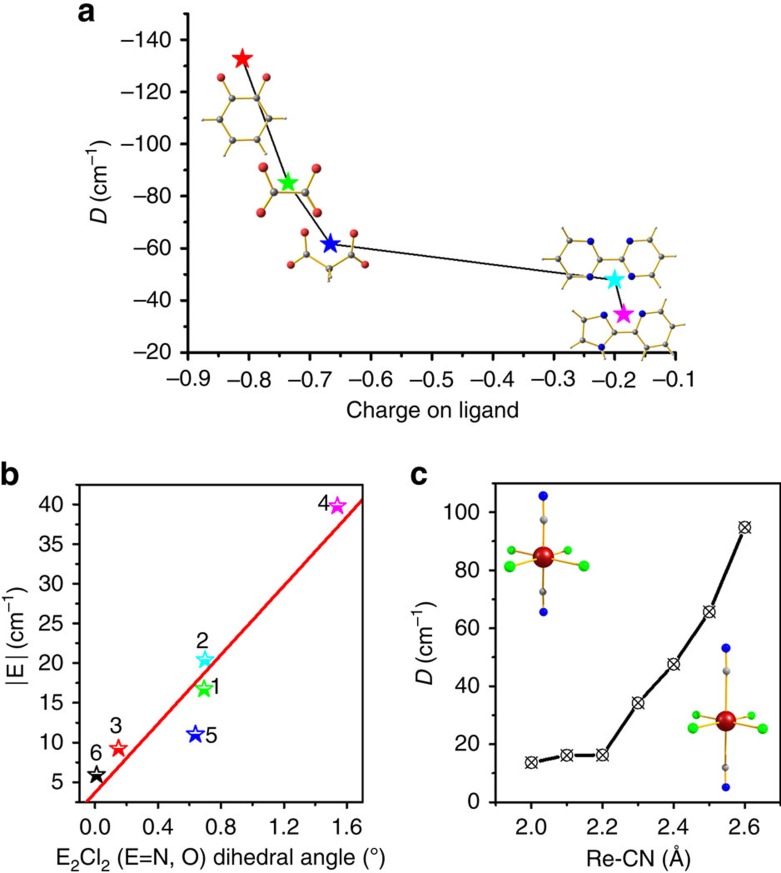
Impact of structural distortions on magnetic anisotropy. (**a**) Plot of computed *D* value versus charge of the coordinated ligand atoms. (**b**) Plot of the computed |E| value versus Re(O/N)_2_Cl_2_ dihedral angle; (**c**) magneto-structural correlation by varying Re–CN bond distances of complex **7**.

The strength of the donor–acceptor abilities significantly affects the magnitude of the *D* values (see Fig. 5 and Supplementary Table 21 for further details)^20^,^62^. In complexes **1**–**6**, larger charge on the donor atoms are found to yield large *D* values (see Fig. 4 and Supplementary Figs 11 and 12 for quantitative charges computed). Larger charges on the donor atoms stabilizes the *d*_*xz*_ orbitals compared with the *d*_*xy*_/*d*_*yz*_ orbitals leading to different transition energies (see Table 1 for details) and thus the computed charges are found to strongly correlated to the magnitude of the *D* values. This striking observation offers a rational approach to fine tune the magnitude of the negative *D* value in this set of complexes. Moreover, stabilization of *d*_*xz*_ orbital also affects the *E* values, as it increases the difference between the *D*_*XX*_ and *D*_*YY*_ contributions leading to a larger *E* with large charge on the ligand (see equation 2 and Supplementary Fig. 12 for details).


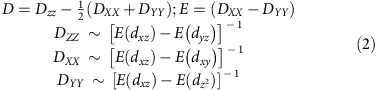


In contrast to complex **7**, where axial positions are occupied by two strong π-acceptor ligands, complex **8** posses two weak π-donor (pyridine) ligands on the axial position and therefore the *d*_*xz*_–*d*_*yz*_ orbital to *d*_*xy*_ orbital is found to be (1,360.7 cm^−1^) much smaller than that of 3,123 cm^−1^ observed in case of complex **7**. Moreover, the first spin-flip-excited state is found at 5,950.5 cm^−1^ (in case of complex **8**), which is again much smaller than the 7,377.3 cm^−1^ gap observed for complex **7** (see Table 1 for details). This leads to larger *D* value for complex **8** compared with complex **7**.

Complexes **9**–**13** possess positive *D* values ranging from +18 cm^−1^ (complex **13**) to +41 cm^−1^ (complex **10**). As the structural parameters across the series are very similar, the differences in the magnitude of the *D* values are expected to arise from the donor strength of the ligand. To affirm this point, we have analysed the donor–acceptor interactions using second-order perturbation theory natural bonding orbitals (NBO) analysis for complexes **10** and **13**. NBO analysis suggests a significant σ-donation from lone pair of nitrogen to Re *d*_*z*^2^_ orbital and this strength is estimated to be 19.6 kcal mol^−1^ for complex **10**, whereas 22.6 kcal mol^−1^ for complex **13** (see Supplementary Fig. 13 for details). Larger σ-donation in complex **13** leads to smaller *D* value compared with complex **10**. A similar analogy can be drawn also for other complexes.

Here independent of the nature of π-donor/acceptor ligands, type I complexes found to yield negative *D* values, whereas type II and III complexes found to yield positive *D* values. This is in stark contrast to the earlier observations where lighter transition metal *d*^3^ ions found to switch the sign of ZFS parameter by changing the nature of the ligand donor atoms (π-donor ligands found to yield +*D* values, whereas π-acceptor ligands yield –*D* values)^66^. This is essentially due to the fact that spin-flip doublet transitions are the dominating factor to the *D* values in Re(IV) complexes, whereas in lighter elements due to smaller crystal-field splitting, the spin-allowed transition dominates the *D* value.

### Magneto-structural *D*-correlations

To probe how the axial bond length influences the *D* value, we have developed a magneto-structural *D* correlations on complex **7** (see Fig. 5c for details), where the axial -CN bonds are varied from 2.0 to 2.6 Å (compression and elongation of axial bonds). As [ReCl_4_(CN)_2_]^2−^ unit has been employed as a building block for synthesis of polynuclear SMMs/SCMs^34^,^35^,^36^,^41^, magneto-structural correlation developed on this model will serve the purpose of obtaining qualitative single-ion Re(IV) anisotropy in diverse polynuclear framework. This Re–C bond distance is found to vary significantly among structures, particularly when the -CN ligands are found to bind to other metal ions. In our correlation, the magnitude of the *D* value found to drastically increase (from +13.6 to +94.7 cm^−1^) as the Re-C bond length increases to 2.7 Å. As the metal–ligand interactions are weaker at longer distances, the transition energies are further lowered leading to larger *D* values for axially elongated structures^62^,^63^. Besides our results reveal that tetragonal distortions does not alter the sign of *D* values and this suggests that the [ReCl_4_(CN)_2_]^2−^ unit unlikely to offer negative single-ion *D* value in any polynuclear framework.

### Mechanism of magnetic relaxation

Complex **1** exhibits field-induced SMM behaviour with a barrier height of 9.6 cm^−1^ at higher temperatures and 1.5 cm^−1^ at lower temperatures. The experimental relaxation observed at higher temperature (up to 3.5 K) is unlikely due to Orbach process as the first excited Kramer's doublet (KD) is estimated to lie at 195 cm^−1^. Thus, the relaxation is expected to be a multi-phonon Raman process. The fast relaxation observed at lower temperature is essentially due to QTM process, which is facilitated due to the presence of transverse anisotropy, hyperfine interactions and external perturbations such as internal magnetic field provided by surrounding molecules. To gain insights into the QTM process, we have analysed the wavefunction of the ground-state KD and our analysis suggests that the ground-state KD comprised of 44% of |3/2,±3/2〉 and 47% of |3/2,±1/2〉. As the *D* value is very large (D>>kT), the |3/2,±1/2〉 KD will be completely depopulated and the ground state can be treated as a pseudo spin 1/2 system. The presence of large *E* term offers a strong mixing between the |3/2,±3/2〉 and |3/2,±1/2〉 components, which allows QTM to facilitate at low temperatures. To qualitatively analyse the mechanism of magnetic relaxation, we have computed the matrix elements between the connected KDs (see Supplementary Fig. 14 and Supplementary Note 3 for details)^67^. Our calculations predict very large tunnelling probability between the ground-state KDs and this is in line with the analysed wave function analysis. Such a prominent QTM process expected to quench the magnetization completely and this is consistent with the absence of zero-field SMM behaviour^56^. On the other hand, application of external d.c. field lifts the degeneracy and suppresses this fast relaxation. However, the QTM process cannot be ignored even under the applied field conditions as hyperfine interactions and intermolecular dipolar couplings facilitate this process. For diluted samples where intermolecular interactions are negligible, hyperfine interactions are the only factor which governs the resonant QTM process^58^,^68^. To gain further insights, we have computed hyperfine interactions of the Re(IV) ions as it has two dominant isotopes ^185^Re and ^187^Re (I=5/2) with a significant natural abundance (see Supplementary Table 22). Particularly, the transverse component of this internal nuclear spin of Re(IV) (measured as |A_X_| and |A_Y_| hyperfine tensors) give rise to a small internal magnetic field inside the molecule facilitating the QTM in zero external field. The hyperfine interactions computed for complexes **1** and **2** are found to be significantly large (1,661, 1,668, 1,669 MHz for complex **1** and 1,962, 1,967 and 1,969 MHz for complex **2**) leading to fast QTM both in the presence and absence of magnetic field. A detailed experimental characterization on the diluted sample needs to be studied to verify the proposed mechanism and to gain further understanding on the relaxation process.

### Role of metal–ligand covalency on magnetic anisotropy

With an aim to analyse the role of metal–ligand covalency on the *D* value of Re(IV) complexes, here we have modelled complexes, [ReCl_4_(E_2_C_6_H_4_)]^2–^ (here E=O (complex **4**), S (**4a**) and Se (**4b**)). For the optimized geometries of **4a** and **4b**, the computed *D* values are +112 and +114 cm^−1^, respectively. Although the strength of the *D* values are only moderately affected, the sign can be predicted unambiguously here as there is a significant drop in the *E*/*D* values (0.30, 0.23 and 0.24 for **4**, **4a** and **4b**, respectively). Besides, the NBO analysis reveals that the Re–S/Se bond is more covalent than Re–O bond (for the Re–O, the Re contribution is 18.4%, whereas oxygen contribution is 81.6% and for Re–S(Se) bond, Re contribution is 32%(36%), whereas the S(Se) contribution is 67% (63%); see Supplementary Figs 15 and 16 and Supplementary Tables 23–25 for details). This difference in covalency lead to larger orbital splitting within t_2g_ sub-shell for S/Se analogues^22^. The larger orbital splitting are compensated by the large SOC associated with S and Se atoms leading to rigorous mixing of excited states with the ground state yielding similar strength of *D* values compared with the oxygen analogue. Interestingly, the π-interactions in **4a** and **4b** stabilize the *d*_*xy*_ and destabilize the *d*_*xz*_/*d*_*yz*_ orbitals leading to a similar strength of *D*_*XX*_ and *D*_*YY*_ contributions. This lead to a decrease in the *E* values for complexes **4a** and **4b** compared with complex **4**.

## Discussion

Here we have probed the origin of contrasting behaviour observed for Re(IV) SMMs where both the giant magnetic anisotropy and fast QTM found to co-exist. The low-lying doublet states are found to govern the sign and magnitude of ZFS parameters in this class of complexes. Our method assessment reveals that pseudo spin approach or effective Hamiltonian approach coupled with CASPT2 calculations needs to be employed to correctly reproduce the sign and magnitude of ZFS parameters. Quite interestingly, the sign of *D* values are found to be predictable based on the coordination mode of the ligands in these complexes where type I complexes found to possess larger negative *D* values, whereas type II and III found to possess a positive *D* values. Nature of the donor ligands as well as charge on the coordinated atoms found to influence only the strength but not the sign of *D* values. Very large hyperfine interactions (both transverse and axial) and rhombic anisotropy computed on these system found to govern the QTM process. By performing additional calculations and by developing magneto-structural correlations, we offer a way to enhance (diminish) the negative *D* (|*E*|) value in these classes of complexes. Some interesting observations are noted where the metal–ligand covalency found to govern the transverse anisotropy, offering a way to quench the inherent fast QTM process in this class of complexes.

## Methods

### *Ab initio* calculations

We have performed the *ab initio* calculations based of wave function theory approach to compute the ZFS in these set of mononuclear complexes. All the calculations have been performed using MOLCAS 7.8 suite of programme^69^. Here we have employed the state average-CASSCF method to compute the ZFS. The active space comprises of three active electrons in five active orbitals (CAS(3,5)). With this active space, we have computed all the 10 quartets and 40 doublet states in the configuration interaction procedure. On top of the converged CASSCF wave function, we have performed MSCASPT2 calculations to treat the dynamical correlations. We have employed ionization potential electron affinity (IPEA) shift of 0.25 to avoid the intruder states problem in CASPT2 calculations. The MS-CASPT2 computed states were further treated in RASSI-SO module, which explicitly computes the spin-orbit states. Furthermore, SINGLE_ANISO module has been utilized on top to compute the reliable spin-Hamiltonian (*D* and g-tensor, orientation of main magnetic axes and main anisotropic axes and local magnetic susceptibility) for each complex. The following ANO-RCC basis sets were used: [8s7p5d3f2g1h.] for Re, [ANO-RCC...5s4p2d.] for Cl, [ANORCC...6s5p3d.] for Br, [ANO-RCC...4s3p2d.] for O, C and N and [ANO-RCC...2s1p] for H during the calculations. The Cholesky decomposition for two electron integral is employed throughout the calculations to save the disk space. Moreover, additional ZFS calculations have been performed using two different techniques: (i) effective Hamiltonian approach and (ii) second-order perturbation method to check out the robustness of reported theoretical methods in predicting correct sign and magnitude of *D* values.

### DFT calculations

Hyperfine interaction of the Re(IV) nuclei were computed within DFT framework, using electron paramagnetic resonance/nuclear magnetic resonance (EPR/NMR) module in the ORCA code^70^. We have employed meta-GGA TPSSH functional along with SARC basis set for the Re, which is much more flexible at core region to estimate all the components of the A-tensors (Fermi Fermi Contact, Spin-dipolar and Spin-orbit coupling) along –*x*, –*y* and –*z* directions. A very tight self consistent field (SCF) (1 × 10^−8^ E_h_) has been kept throughout the calculations.

## Additional Information

**How to cite this article:** Singh, S. K. & Rajaraman, G. Deciphering the origin of giant magnetic anisotropy and fast quantum tunnelling in Rhenium(IV) single-molecule magnets. *Nat. Commun*. 7:10669 doi: 10.1038/ncomms10669 (2016).

## Supplementary Material

Supplementary Information Supplementary Figures 1-16, Supplementary Tables 1-25 and Supplementary Notes 1-3

## Figures and Tables

**Table 1:** MS-CASPT2+RASSI-SO computed *D* and |*E*/*D*| value for all studied Re(IV) mononuclear complexes along with first spin-free excitation energy.

Complex	*D*_cal_	|*E*/*D*|_cal_	*D* (|*E*/*D*|)_exp_	Δ*E*	References
**1** [ReBr_4_(ox)]^2–^	−93.0	0.18	−73 (0.20)*	8,079.2	39
**2** [ReCl_4_(ox)]^2–^	−85.0	0.24	−57 (0.26)*	8,873.5	39
**3** [ReCl_4_(mal)]^2–^	−61.6	0.15	55†	8,442.8	44
**4** [ReCl_4_(cat)]^2–^	±132.6	0.30	95†	7,526.7	50
**5** [ReCl_4_(bpym)]^2–^	−47.9	0.23	—	7,616.5	46
**6** [ReCl_4_(pyim)]^2–^	−34.7	0.17	—	7,804.7	48
**7** [ReCl_4_(CN)_2_]^2–^	+16.2	0.23	+11 (0.29)*	7,377.3	41
**8** [ReCl_4_(py)_2_]	+55.6	0.18	9.56†	5,950.5	43
**9** [ReCl_5_(py)]^–^	±32.7	0.31	3.52†	7,841.7	51
**10** [ReCl_5_(pyd)]^–^	+41.0	0.24	14.1†	7,385.9	47
**11** [ReCl_5_(pym)]^–^	+24.6	0.17	6.2†	8,300.7	47
**12** [ReCl_5_(pyz)]^–^	+32.5	0.20	9.4†	7,630.2	49
**13** [ReCl_5_(dmf)]^–^	+18.5	0.18	10.1†	8,467.4	45

bpym, 2,2′-bipyrimidine; cat, catechol; dmf, dimethyl formamide; mal, malonato; ox, oxalato; pyim, 2-(2′-pyridyl)imidazole; py, pyridine; pyd, pyridazine; pym, pyrimidine; pyz, pyrazine.

All the *D* values and spin-free excitations energies are provided in cm^−1^.

^*^HF-EPR reported values.

^†^Obtained from magnetic susceptibility measurements, no sign convention has been used.
