# Supplementary material for: Deciphering the origin of giant magnetic anisotropy and fast quantum tunnelling in Rhenium(IV) single-molecule magnets
Source: Nat Commun. 2016 Feb 17;7:10669. doi: 10.1038/ncomms10669 (PMC4757791; doi:10.1038/ncomms10669)
Supplement: Supplementary Information — Supplementary Figures 1-16, Supplementary Tables 1-25 and Supplementary Notes 1-3 [file ncomms10669-s1.pdf]

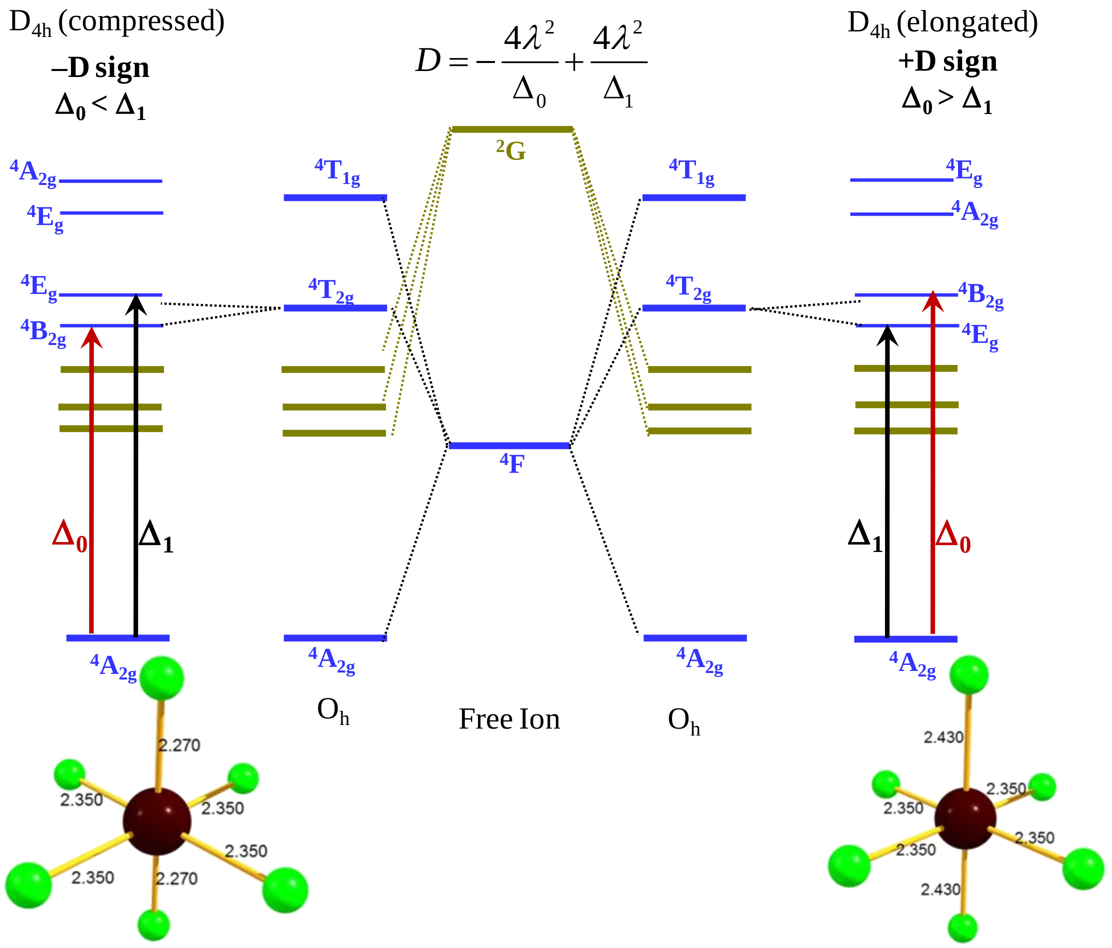

Supplementary Figure 1. Splitting pattern of the low-lying spin free states of d^3 ion under tetragonal distortion (axial compression and axial elongation).

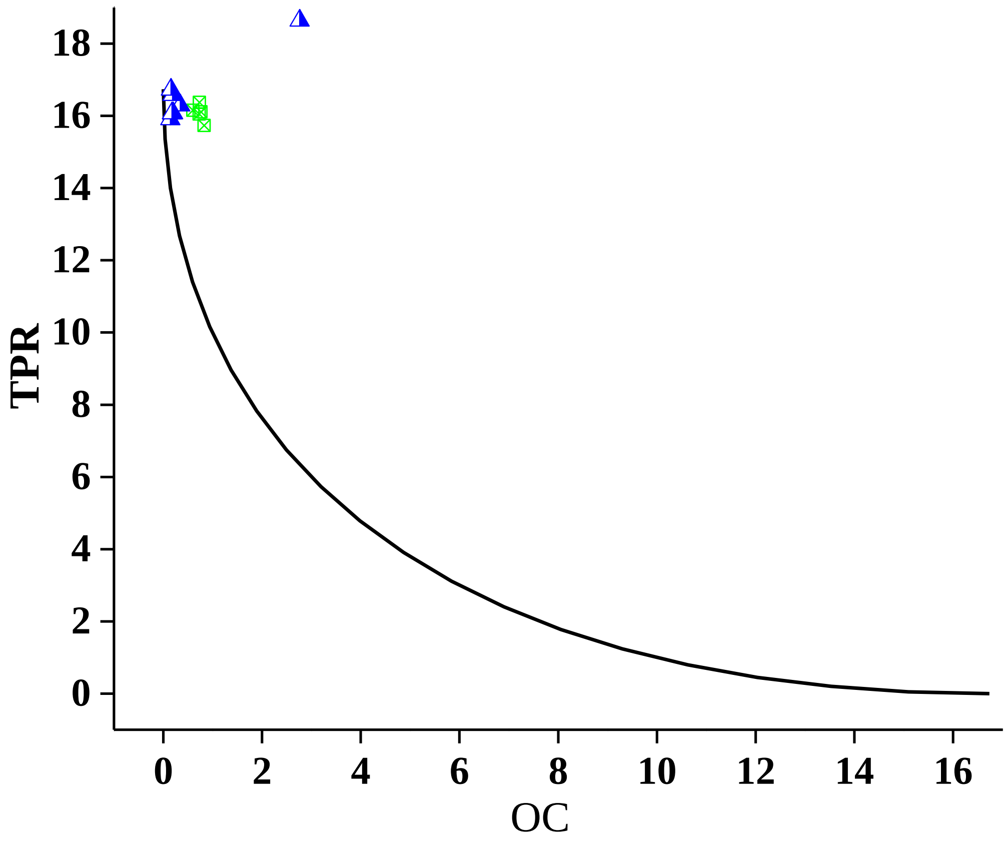

Supplementary Figure 2. Continuous symmetry map for the studied thirteen Re(IV) complexes. On the y-axis the TPR represents trigonal prismatic, on x-axis the OC represents octahedron geometry. Green hollow squares represent the complexes **1-6**, blue half filled triangles represent complexes **7-13**. The thick black line represents the minimal distortion pathway from octahedron to trigonal prismatic shape.

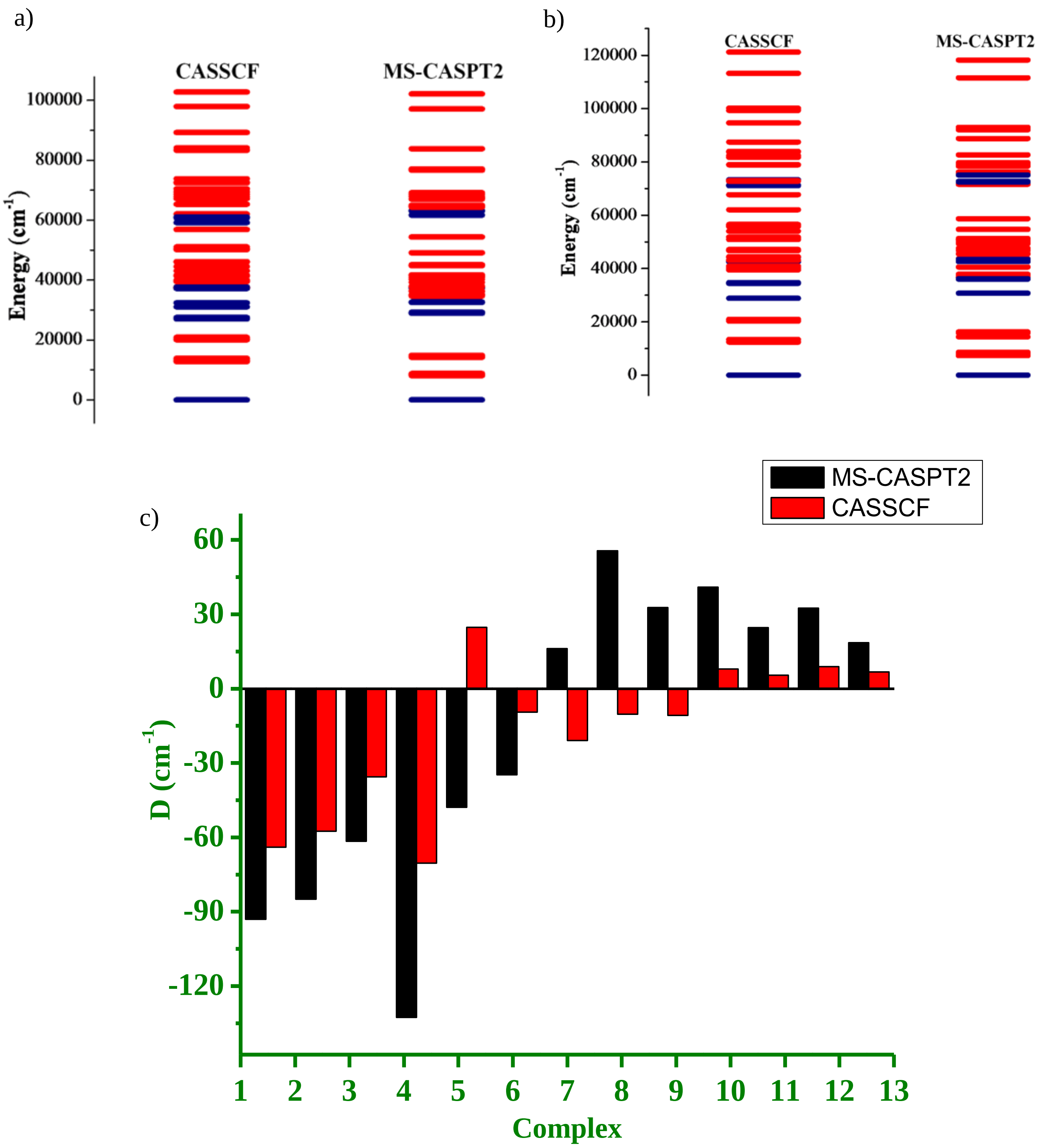

Supplementary Figure 3. CASSCF and MS-CASPT2 computed 10 quartet and 40 doublet states for complex **1** and complex **7**. The blue thick lines represent quartet states while red thick lines represent doublet states. a) for complex **1**; b) for complex **7** c) MS-CASPT2 and CASSCF computed D values for all the complexes.

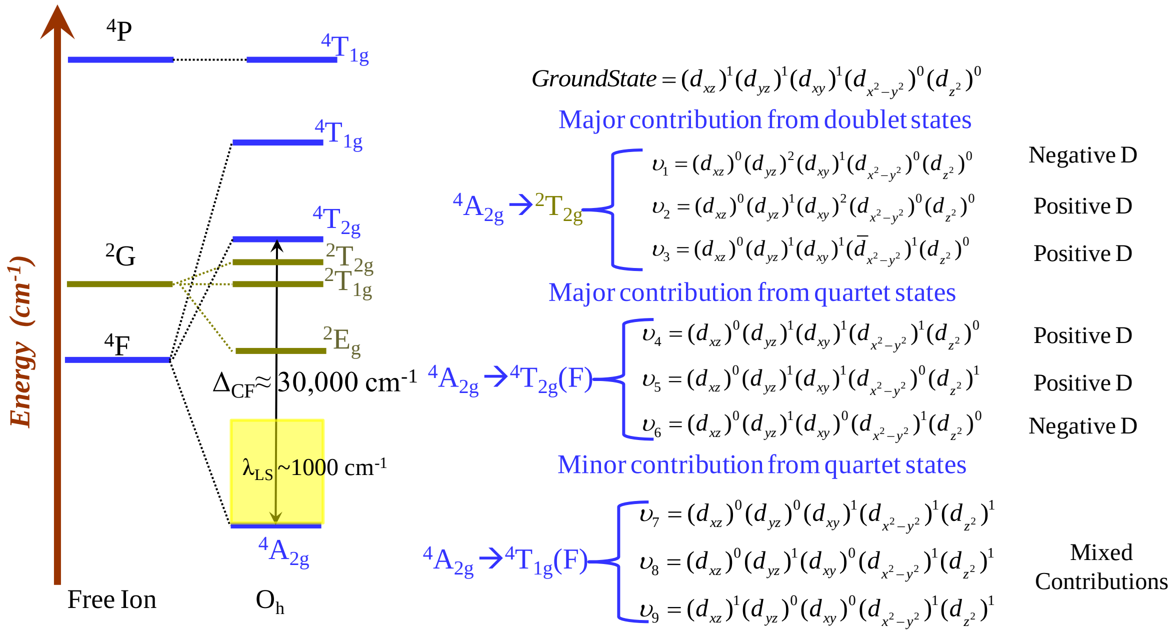

Supplementary Figure 4. Splitting pattern of the Re(IV) ion in the octahedral ligand field. We have also marked the important spin-conserved and spin-flip excitations which are major contributor to the D values. Here υ represent the excitations and labelled number of excitations. Here the bar sign of the d orbital representing spin-down.

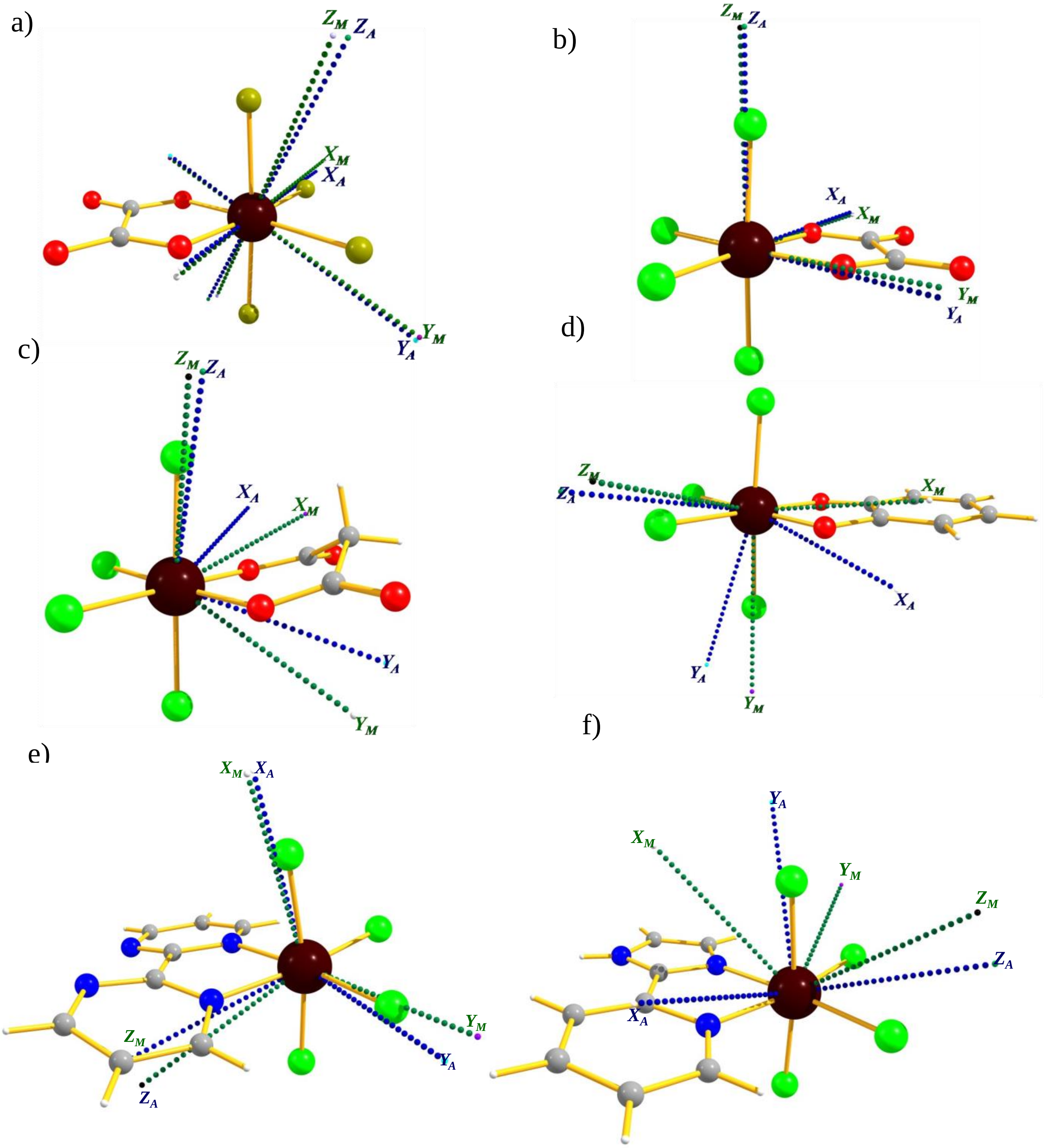

a)
Z_M Z_A
X_M
X_A
Y_M
Y_A
b)
Z_M Z_A
X_A
X_M
Y_M
Y_A
c)
Z_M Z_A
X_A
X_M
Y_A
Y_M
d)
Z_M
Z_A
X_M
X_A
Y_A
Y_M
e)
X_M X_A
Y_M
Y_A
Z_M
Z_A
f)
Y_A
X_M
Y_M
Z_M
Z_A
X_A

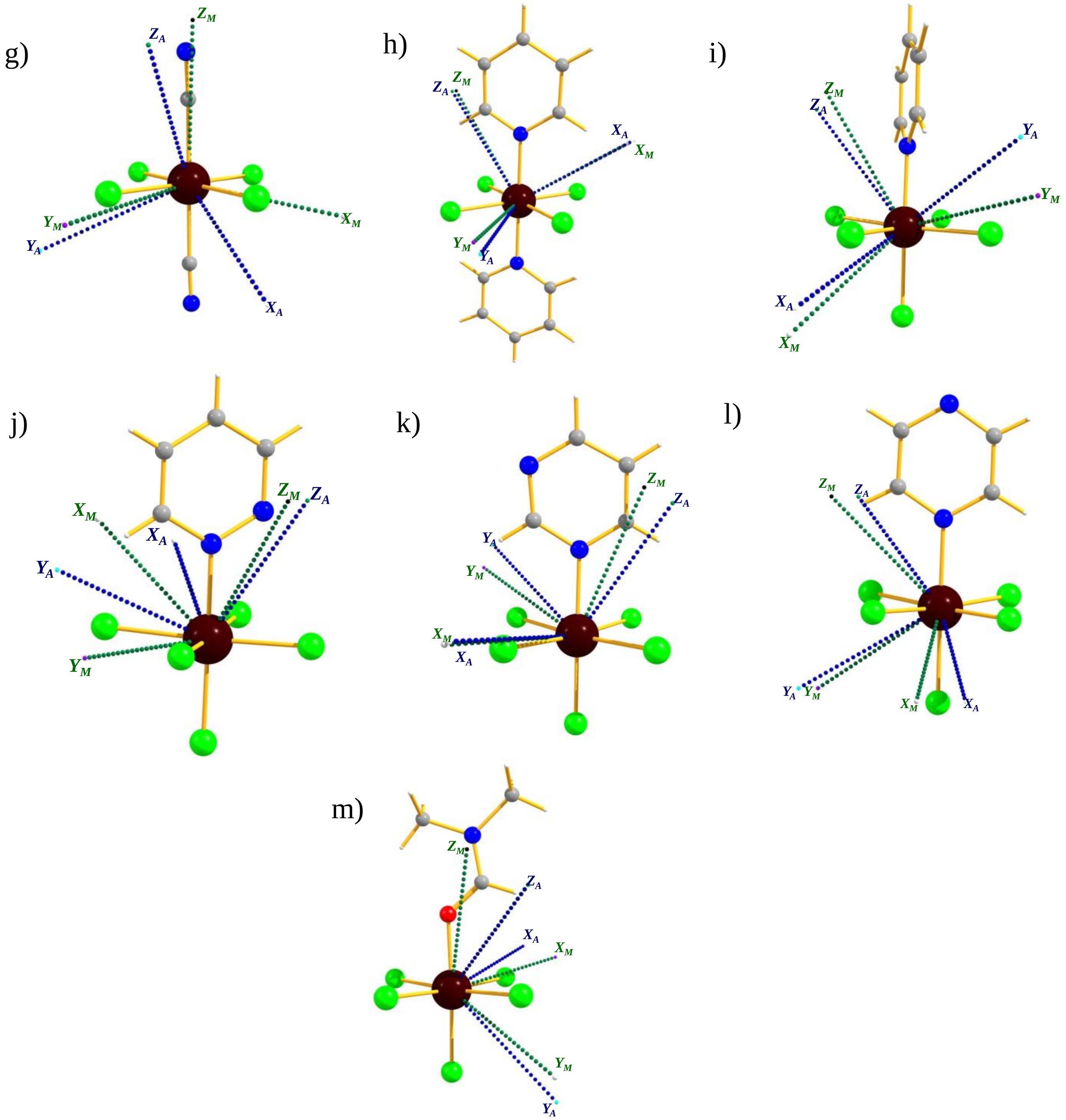

Supplementary Figure 5. CASPT2+RASSI computed orientation of the main magnetic axes (X_M, Y_M and Z_M) and main anisotropy axes (X_A, Y_A and Z_A) representing axes of g-tensor and D-tensor respectively for all the thirteen complexes. a)**1**; b) **2**; c) **3**; d)**4** ; e)**5**; f)**6**; g)**7**; h) **8**; i); **9**; j) **10**; k)**11**; l)**12**; m)**13**. Color Code: dark brown, Re; pale brown, Br; light green, Cl; blue, N; grey, C; white, H. Light blue colour plane associated with four atoms represents the equatorial plane.

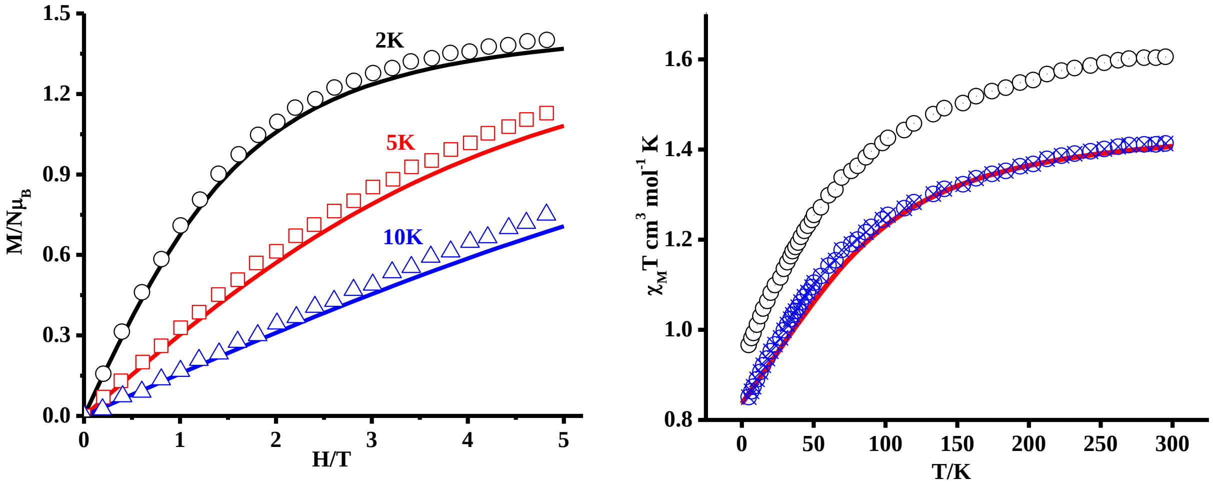

Supplementary Figure 6. Field dependence magnetization data and Thermal variation of $\chi_M T$ for complex **1**. a) Field dependence magnetization data at different temperatures. The black hollow circles, red squares and blue triangles are the experimental magnetization data for complex **2** at 2K, 5K and 10 K respectively while the corresponding coloured lines are the RASSI-SO computed powder magnetization data; b) Thermal variation of $\chi_M T$ complex **1,** where hollow black dotted circles are the experimental data obtained from the experimental plot. The red line represents the RASSI-SO computed thermal variation of the $\chi_M T$. The blue crossed circles represent the experimental magnetic susceptibility, scaled by factor 0.88. All the experimental data were digitized from the experimental plot.

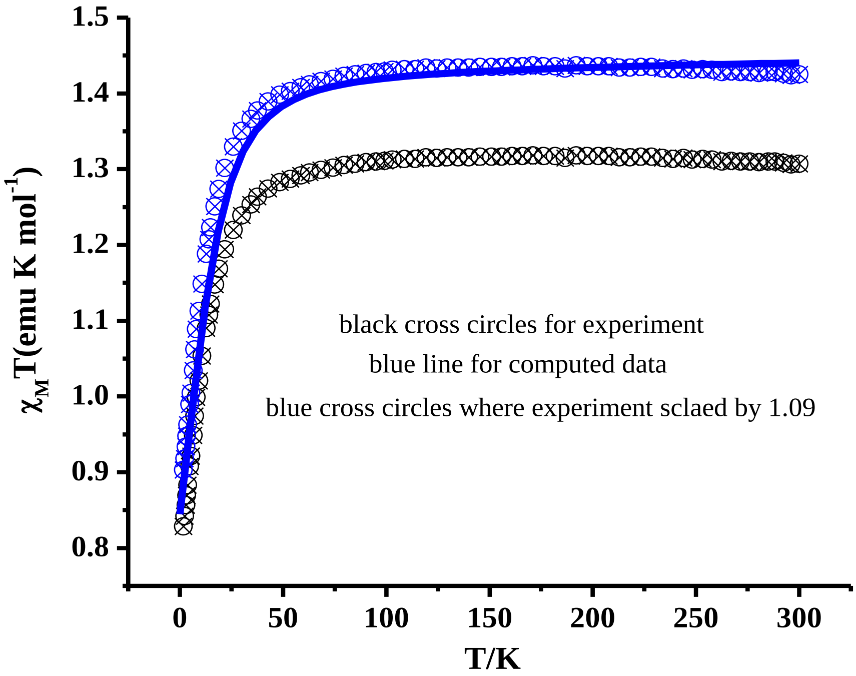

Supplementary Figure 7. Thermal variation of χ_MT complex **7**, where hollow black cross circles are the experimental data obtained from the experimental plot. The blue line represents the RASSI-SO computed thermal variation of the χ_MT. The blue crossed circles represent the experimental magnetic susceptibility, scaled by factor 1.09. All the experimental data were digitized from the experimental plot

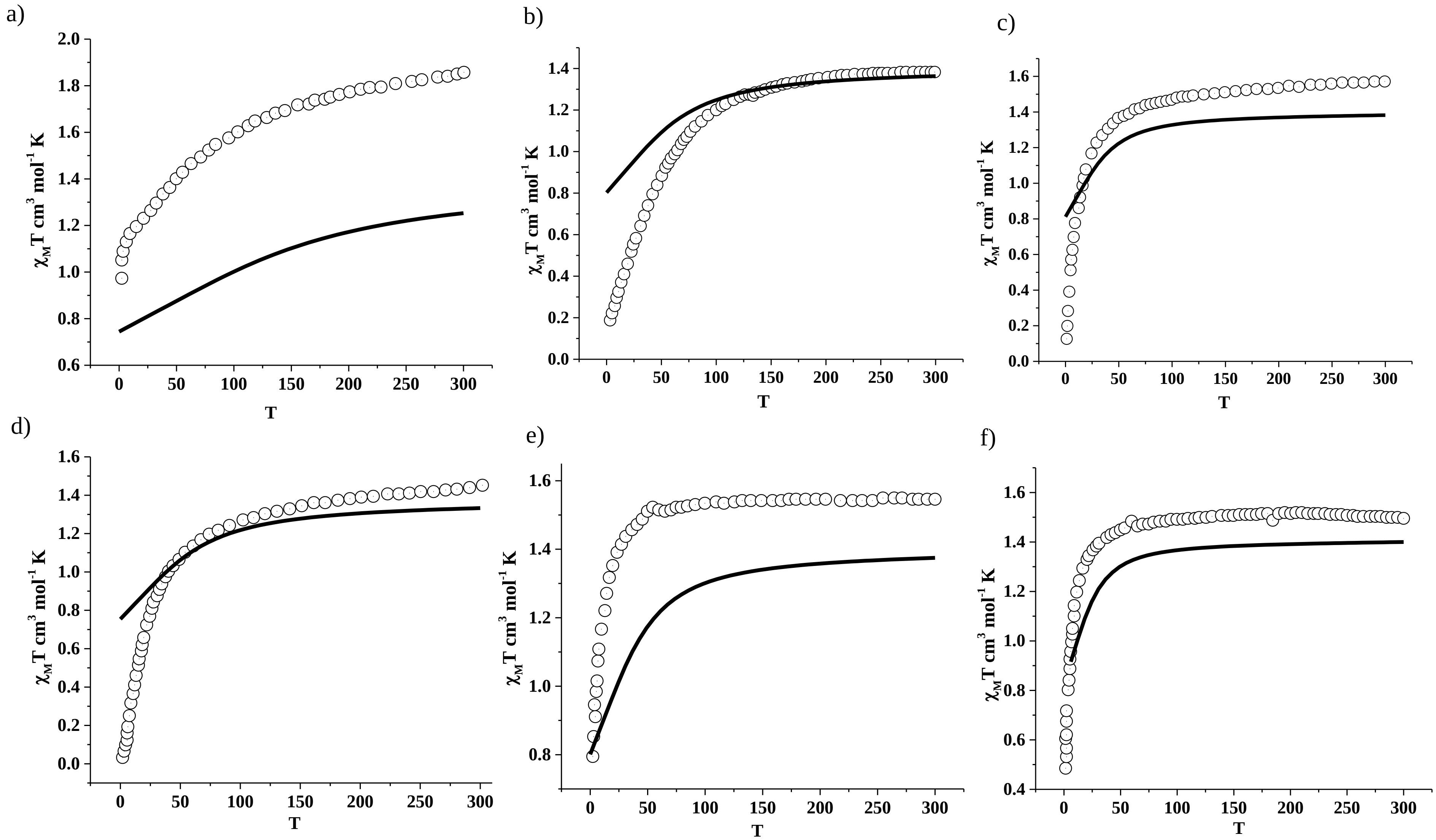
a)
b)
c)
d)
e)
f)
χMT cm3 mol-1 K
T
0
50
100
150
200
250
300

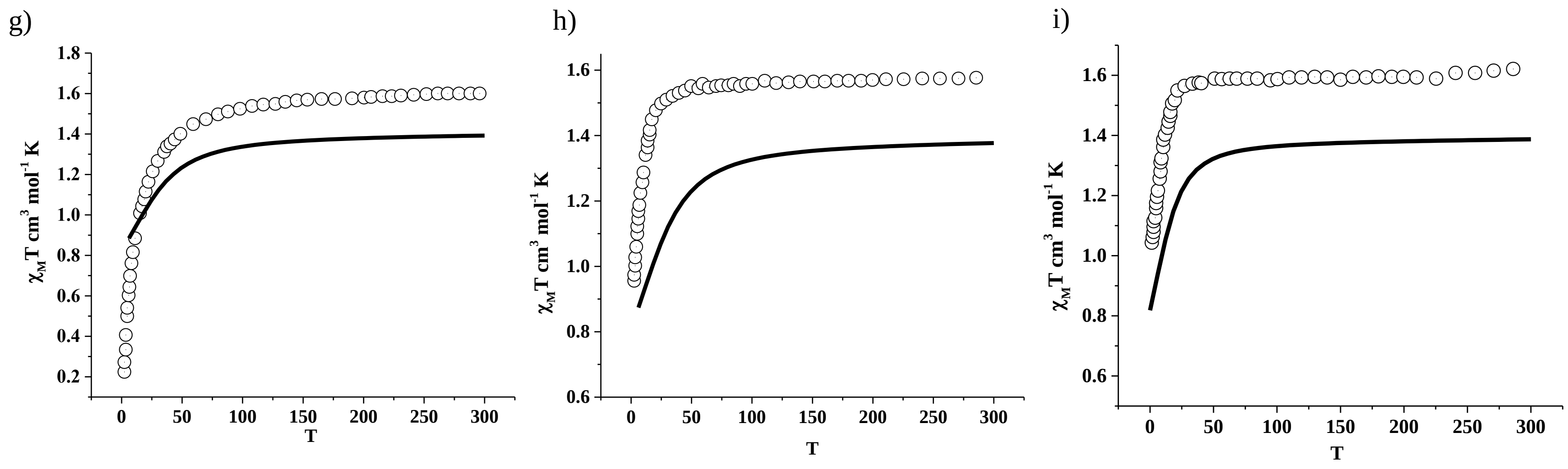

Supplementary Figure 8. Computed and Experimental thermal variation of χ_MT for nine complexes. The hollow black circles are the experimental data digitized from experimental plot while the black thick lines are computed data. a)$[ReCl_4(cat)]^{2-}$];b) $[ReCl_4(mal)]^{2-}$;c) $[ReCl_5(py)]^{-}$; d) $[ReCl_4(py)_2]$; e) $[ReCl_5(pyd)]^{-}$; f) $[ReCl_5(pym)]^{-}$; g) $[ReCl_4(pyim)]$; h) $[ReCl_5(pyz)]^{-}$; i) $[ReCl_5(dmf)]^{-}$

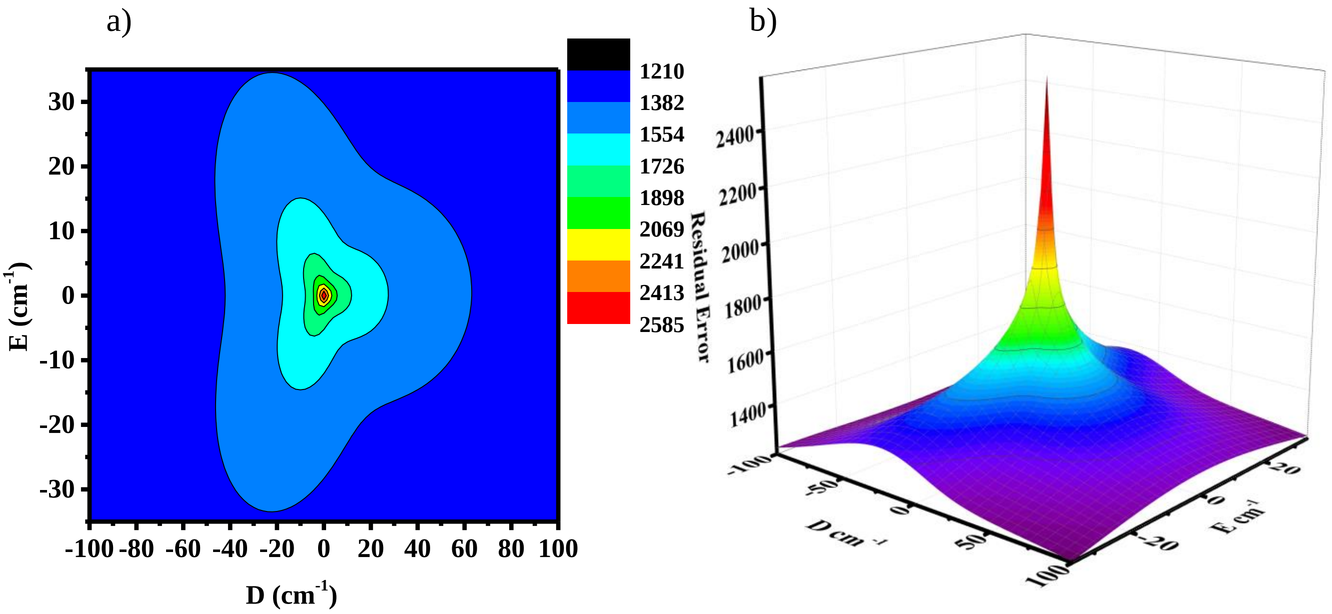

Supplementary Figure 9. Residual error plot for complex **1**; a) contour plots showing the residual error of susceptibility and magnetization curves when varying D and E from -100 to +100 cm^{-1} and -30 to +30 cm^{-1}, respectively, with fixed g_{xx}, g_{yy} and g_{zz} values of 1.726, 1.712 and 1.698. The dark blue colour signifies the minimum residual error for fitting the magnetisation and susceptibility together; b) with similar pattern 3D surface has been created where X and Y axis shows E and D values with Z axis as residual error. Here the violet region signifies the minimum residual error.

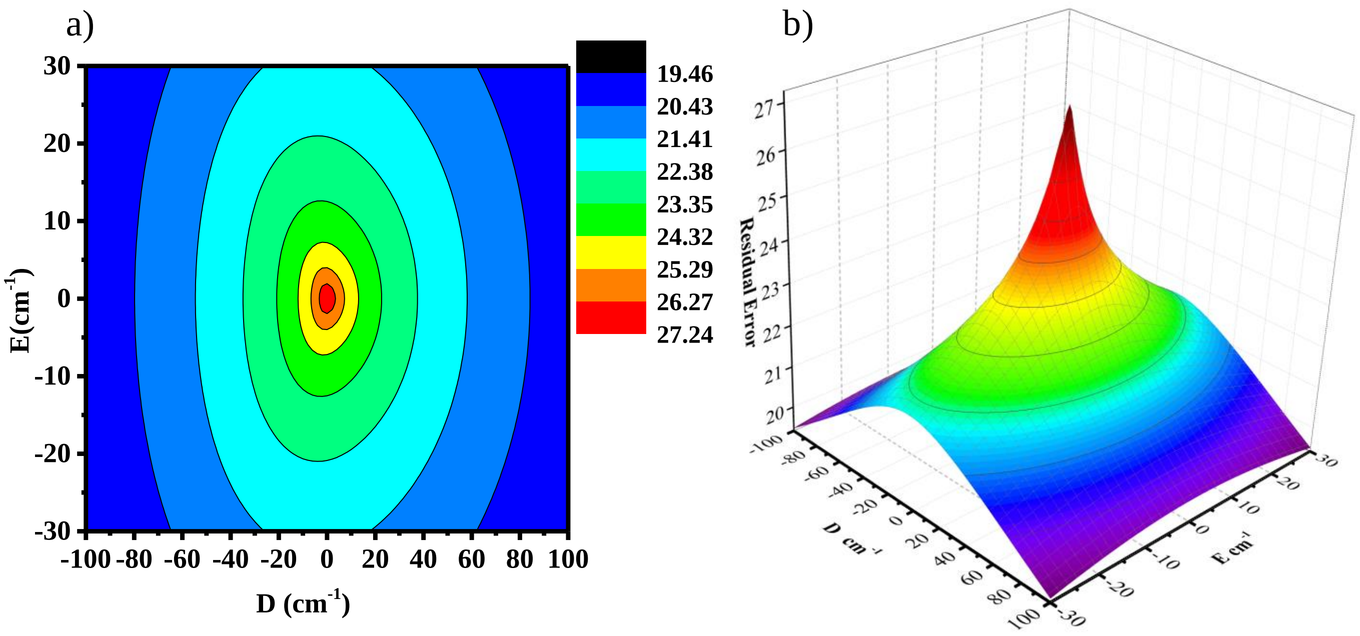

Supplementary Figure 10. Residual error plot for complex **7**; a) contour plots showing the residual error of susceptibility only when varying D and E from -100 to +100 cm^{-1} and -30 to +30 cm^{-1}, respectively, with fixed g_{xx}, g_{yy} and g_{zz} values of 1.751, 1.756 and 1.729. The dark blue colour signifies the minimum residual error for fitting the magnetisation and susceptibility together; b) with similar pattern 3D surface has been created where X and Y axis shows E and D values with Z axis as residual error. Here the violet region signifies the minimum residual error.

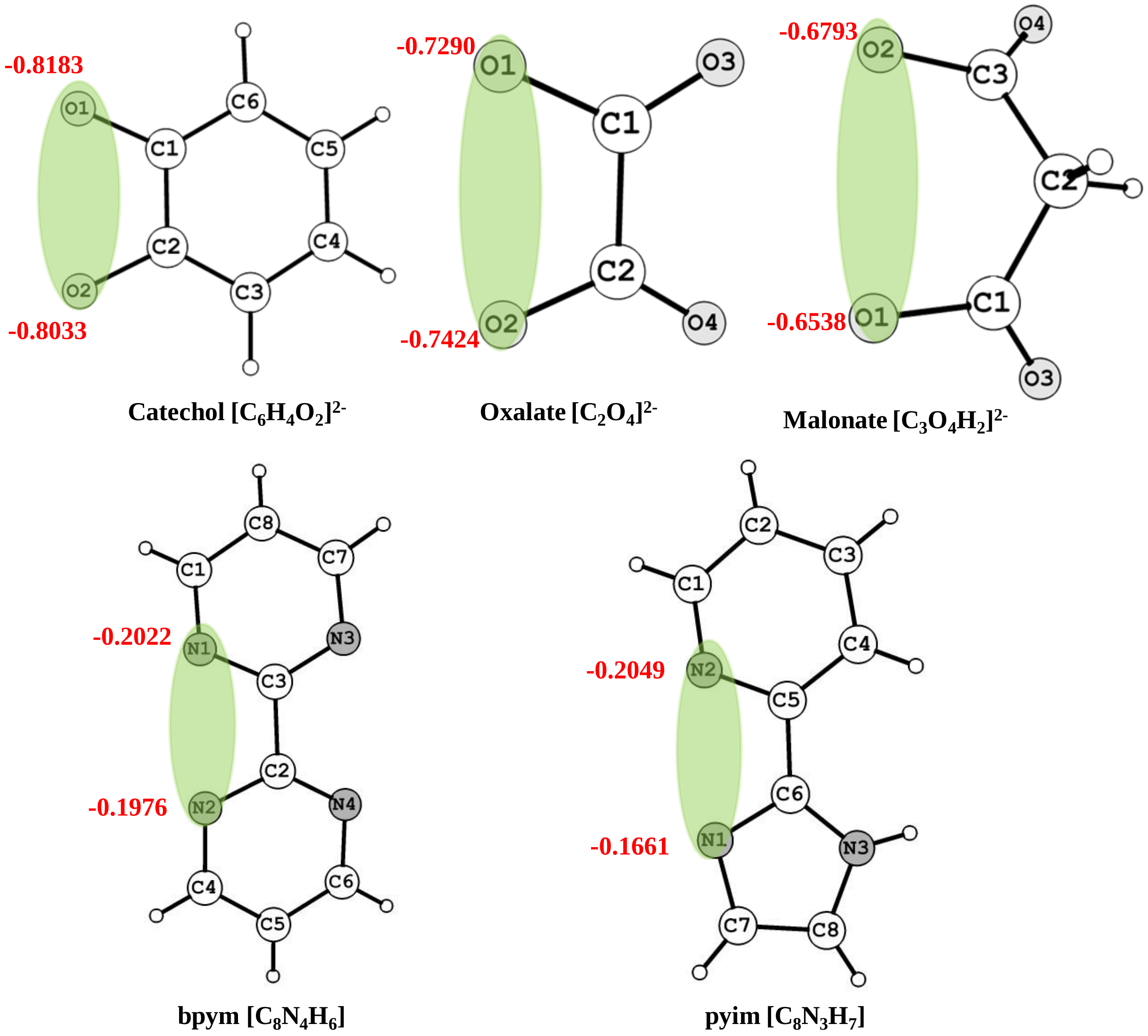

Supplementary Figure 11. MS-CASPT2 computed Mulliken atomic charges on oxalate, malonate, catechol, pyridimide, 2-(2’-pyridyl) biimidazole ligands. The marked light green colour shows the coordinating mode. The charges here shown for the coordinating atoms.

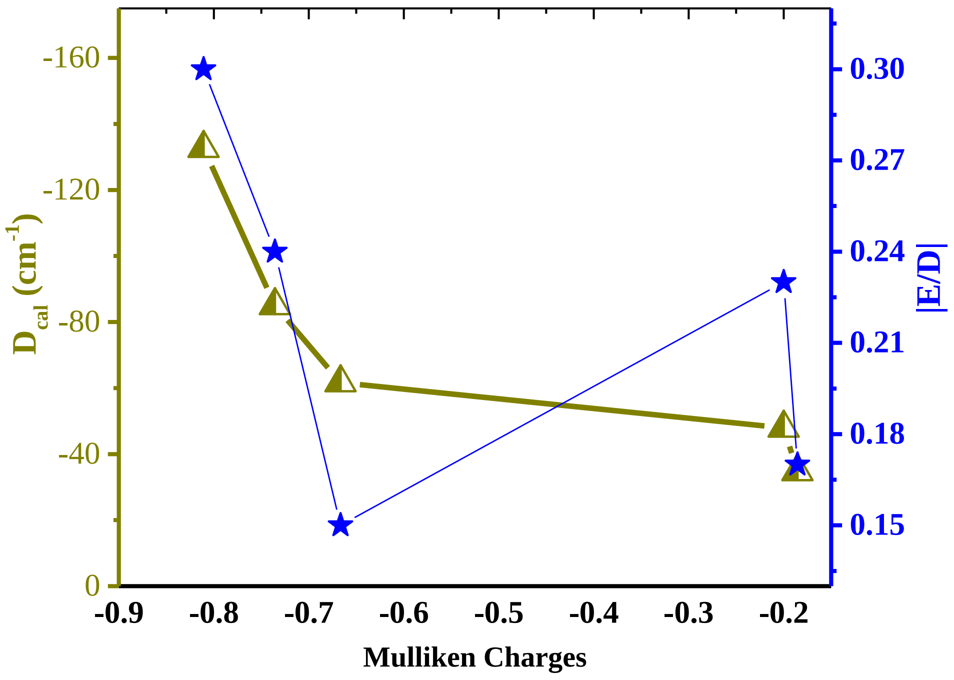

Supplementary Figure 12. Qualitative trend observed between MS-CASPT2 computed D, E/D values and MS-CASPT2 charge on the equatorial coordinated ligand. The black thick line in the centre represents the limit of the rhombicity.

N(2p_x)→ 5d type antibonding orbital of Re-Cl bond
E = 19.64 kcal/mol

O(2p_x)→ 5d type antibonding orbital of Re-Cl bond
E = 22.64 kcal/mol

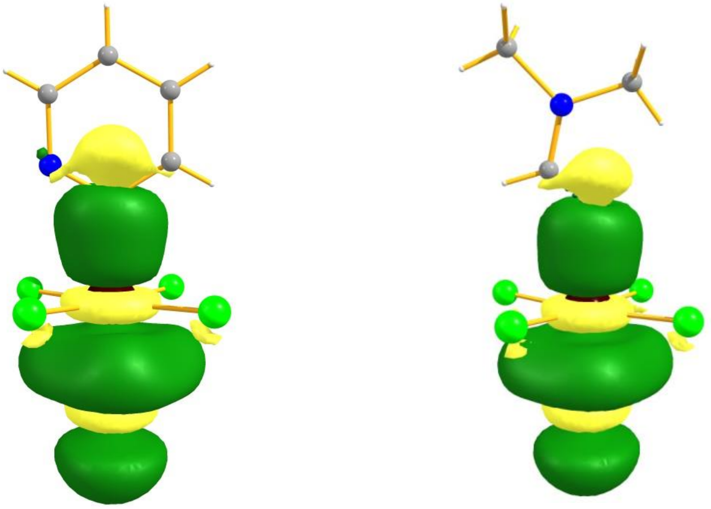

Complex 10 **Complex 13**

Supplementary Figure 13. Donor acceptor interaction computed using second order perturbation theory NBO analysis.

.

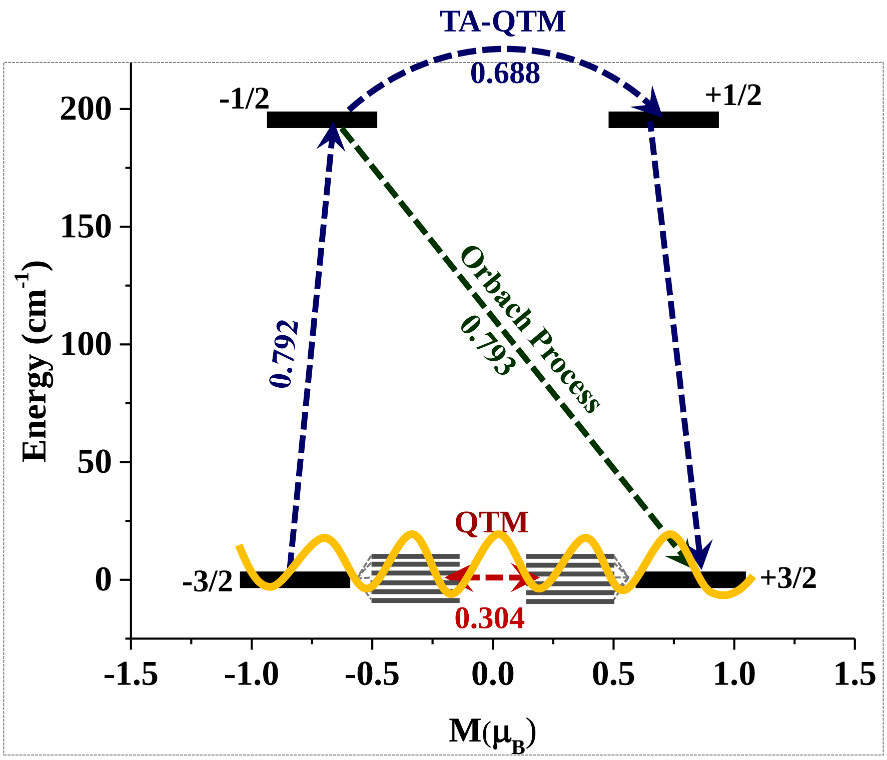

Supplementary Figure 14. MS-CASPT2/RASSI-SO computed *ab initio* blockade barrier for magnetic relaxation.

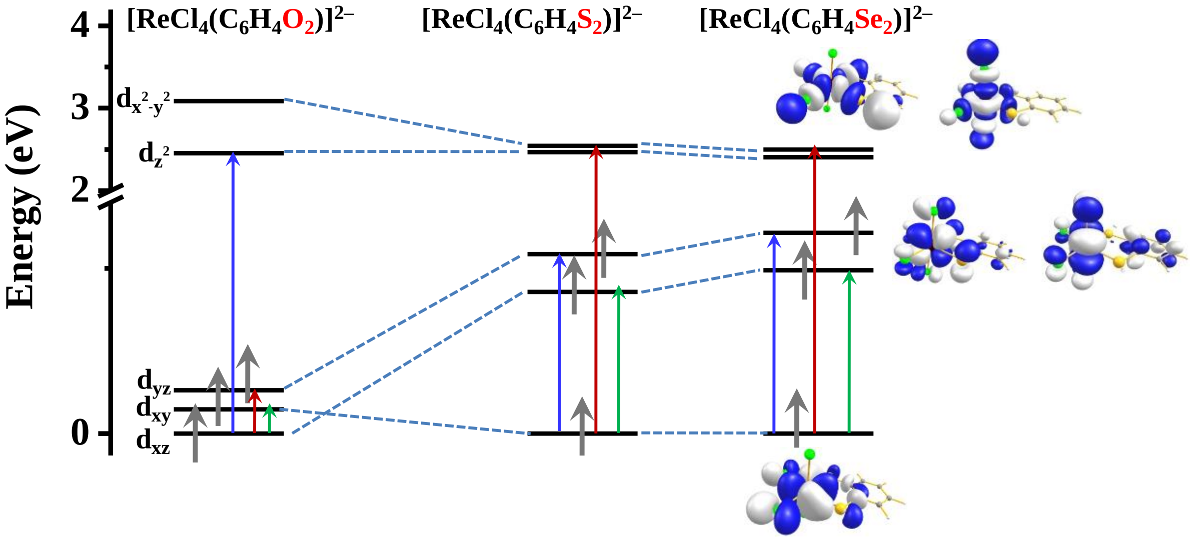

Supplementary Figure 15. Crystal field splitting pattern of d-orbitals of Re(IV) complexes in the $[ReCl_4(C_6H_4O_2)]^{2-}$ complex (**4**), $[ReCl_4(C_6H_4S_2)]^{2-}$ complex (**4a**) and $[ReCl_4(C_6H_4Se_2)]^{2-}$ complex (**4b**) The red, green and blue arrows represent the D_{ZZ}, D_{XX} and D_{YY} contributions, respectively.

Nature of excitations in complex **4**.

$$D_{ZZ} \sim [E(d_{xz}) - E(d_{yz})]^{-1}$$
$$D_{XX} \sim [E(d_{xz}) - E(d_{xy})]^{-1} \quad (1)$$
$$D_{YY} \sim [E(d_{xz}) - E(d_{z^2})]^{-1}$$

Nature of excitations in complex **4a** and **4b**.

$$D_{ZZ} \sim [E(d_{xy}) - E(\bar{d}_{x^2-y^2})]^{-1}$$
$$D_{XX} \sim [E(d_{xy}) - E(d_{xz})]^{-1} \quad (1)$$
$$D_{YY} \sim [E(d_{xy}) - E(d_{yz})]^{-1}$$

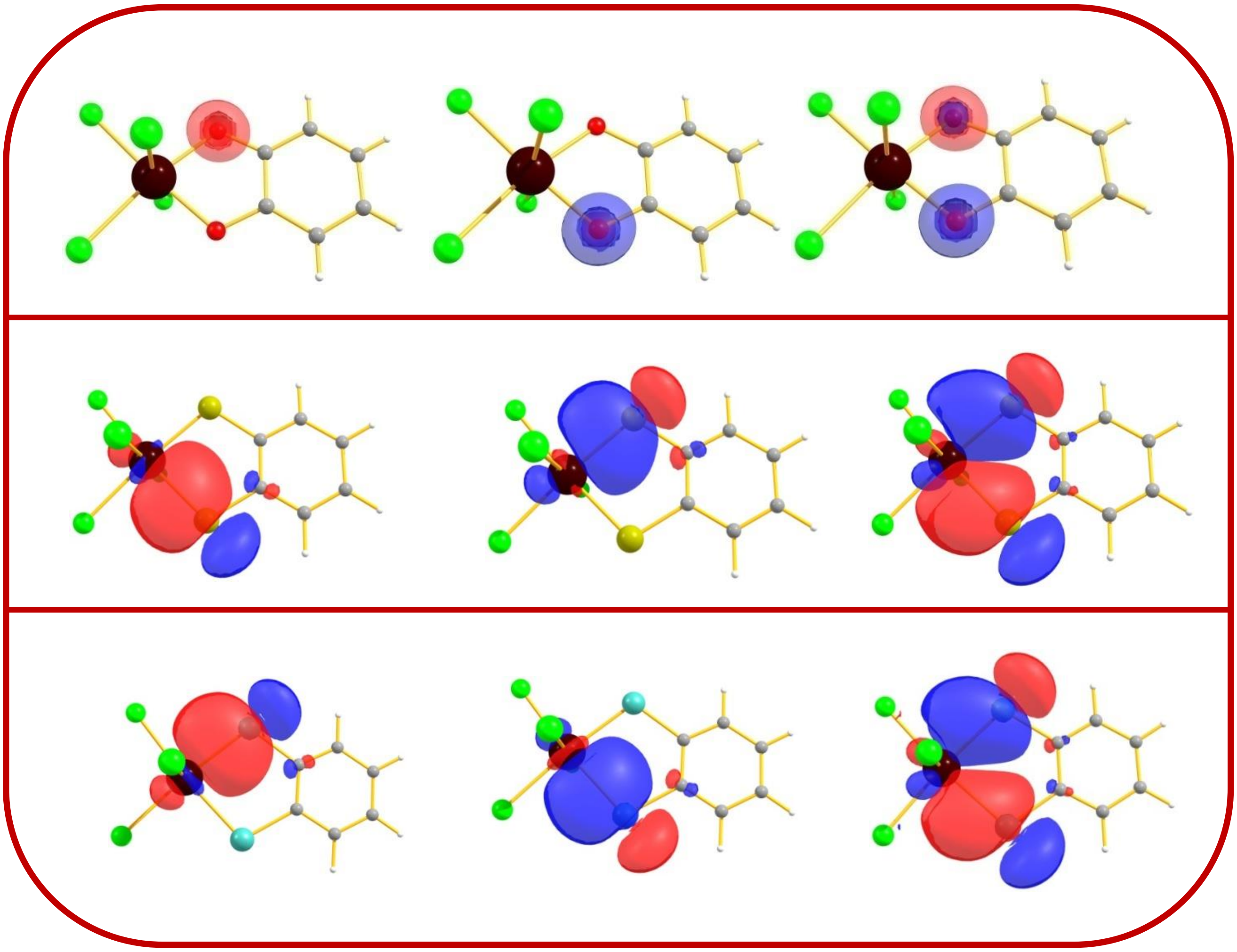

Supplementary Figure 16. DFT computed σ-bonding NBO for complexes **4**, **4a** and **4b** representing the nature of Re-E (where E = O(**4**), S(**4a**) and Se(**4b**)) bonds. Top section represents the Re-O, middle section represents Re-S and the last section represents the Re-Se bonds. First two orbitals of each section represents the individual contributions, while the third picture has been created where both the NBO's are subtracted and plotted together for better visualisation.

Supplementary Table 1. MS-CASPT2 computed first three low-lying energies, D and g values for model complexes elongated D_{4h} $[ReCl_6]^{2-}$ and compressed D_{4h} $[ReCl_6]^{2-}$

Energies of KDs (cm^{-1})	Energies of three-low lying spin-free states			Computed D values@MS-CASPT2 for elongated D_{4h} $[ReCl_6]^{2-}$
0.000<br>39.146	$^4A_2$		0.000	D = +19.3 cm^{-1}<br>|E| = 1.97 cm^{-1}
	$^4T_{2g}$	^4E_g	27888.5<br>27903.4	
		$^4B_{2g}$	30226.6	g_X = 1.6960, g_Y = 1.6978, g_Z = 1.7178
	Spin-free energy			Computed D values@MS-CASPT2 for compressed D_{4h} $[ReCl_6]^{2-}$
0.000<br>48.923	$^4A_2$		0.000	D = -24.3 cm^{-1}<br>|E| = 1.67 cm^{-1}
	$^4T_{2g}$	$^4B_{2g}$	30008.7	
		^4E_g	31033.1<br>31049.9	g_X = 1.7352, g_Y = 1.7335, g_Z = 1.7123

Supplementary Table 2. SHAPE computed minimal distortion from the ideal geometry in hexa coordination. OC stands for octahedron and TPR stands for the trigonal prismatic.

Complex	OC	TPR
**1**	0.72707	16.05207
**3**	0.60109	16.15357
**4**	0.82902	15.73304
**5**	0.73341	16.37012
**6**	0.76192	16.10946
**7**	2.76214	18.63793
**8**	0.17456	16.06944
**9**	0.15434	16.7269
**10**	0.19784	16.07452
**11**	0.18836	16.57904
**12**	0.14481	15.90824
**13**	0.3438	16.29249

Supplementary Table 3. Selected X-ray crystal structural parameters for complexes **1-13**

Complex	avg. Re-Cl_{ax}	avg. Re-Cl_{eq}	avg. $\angle Cl_{eq}$-Re-Cl_{eq}	avg. Re-L_{eq}	avg. Re-L_{ax}	$\angle L_{eq}$-Re-L_{eq}	$\angle Cl_{ax}$-Re-Cl_{ax} or $\angle L_{ax}$-Re-L_{ax} or $\angle L_{ax}$-Re-Cl_{ax}
	(Å)	(Å)	(°)	(Å)	(Å)	(°)	(°)
**1**	2.5028	2.4790	93.9	2.0212	---	79.7	176.6
**2**	2.3536	2.3305	94.1	2.0321	---	79.9	177.0
**3**	2.3558	2.3351	93.9	2.0077	---	87.1	176.4
**4**	2.3621	2.3410	94.8	2.0076	---	79.4	175.2
**5**	2.3326	2.3111	94.8	2.1322	---	76.3	170.5
**6**	2.3394	2.3191	96.6	2.1166	---	76.3	173.3
**7**	---	2.3367	90.1	---	2.1330	---	180
**8**	---	2.3303	89.7	---	2.1427	---	180
**9**	2.3356	2.3505	89.3	---	2.1447	---	177.7
**10**	2.3194	2.3385	90.04	---	2.1234	---	178.2
**11**	2.3131	2.3436	89.6	---	2.1702	---	179.2
**12**	2.3203	2.3423	89.6	---	2.1732	---	178.5
**13**	2.3167	2.3501	89.9	---	2.0630	---	176.9

.

Supplementary Table 4. CASSCF+RASSI computed spin-free and spin-orbit states for $[ReBr_4(ox)]^{2-}$ (**1**)

Spin-free Energies		Spin-Orbit Energies	
CASSCF	MS-CASPT2	CASSCF	MS-CASPT2
0	0	0	0
27027.194	28878.313	136.961	195.442
27532.067	29262.949	12012.709	7482.693
31071.134	32523.404	12277.343	7867.618
32386.975	32643.335	13131.434	8874.822
37186.085	37193.625	13408.171	9039.951
37639.602	37289.791	14244.529	9521.821
59187.66	61717.128	21332.057	15695.799
60752.765	63058.781	22172.026	17043.378
61095.22	63253.994	22506.007	17542.606
12791.535	8079.273	26944.193	28569.545
12862.232	8150.766	27666.194	29164.934
12999.809	8274.809	28233.363	29618.852
13414.235	8384.318	29340.071	30897.706
13862.231	8762.708	31412.242	32763.087
20051.091	14221.753	31920.485	33102.525
20440.894	14314.114	33622.786	33407.228
20860.83	14843.674	33935.487	34146.723
37328.176	34707.22	36653.4	35048.93
37596.158	34913.341	37418.027	35907.624
37727.905	35223.757	37728.537	36516.837
39495.558	36223.994	38303.381	37280.392
39747.23	36314.432	38832.132	37505.888
40023.793	36357.541	39808.621	37972.472
41306.756	37844.875	40437.61	38427.524
41672.215	37919.843	40789.996	38792.003
43138.075	39288.59	41120.307	38987.893
44508.007	40208.594	42079.848	39681.514
46059.972	40871.185	42188.677	39984.501
46117.682	41489.446	43737.112	40570.548
46178.406	41716.838	44589.822	41131.457
50233.799	44787.217	45649.193	42235.096
50479.324	44981.432	46935.27	42509.163
51076.601	45159.624	47217.288	43242.529
56902.917	49124.205	48615.448	44460.237
62039.534	54420.512	51274.452	46531.44
65327.209	64347.06	52055.867	46826.487
67181.219	64832.732	52598.567	47205.233
67865.599	64984.441	58396.122	51574.585
68686.618	67105.474	60281.854	56770.514
69483.87	67154.884	60389.007	62770.2
70519.068	67870.518	62001.057	63041.474
72448.752	68554.202	62843.134	64063.547
72711.913	69151.123	62943.442	64780.733
73822.163	69184.152	64106.481	64947.288
83378.377	76670.815	64364.464	65284.86
84119.852	77109.155	66615.458	66089.729
89276.123	83846.102	68695.245	66885.598
98038.814	97190.456	69355.834	67181.379
102841.886	102159.951	71012.281	68974.237
		71449.033	69439.205
		72155.158	70484.902
		73688.362	70991.295
		74929.82	72162.983
		76561.201	72452.312
		....	....

Supplementary Table 5. CASSCF+RASSI computed spin-free and spin-orbit states for $[ReCl_4(ox)]^{2-}$ (**2**)

Spin-free Energies		Spin-Orbit Energies	
CASSCF	MS-CASPT2	CASSCF	MS-CASPT2
0	0	0	0
29244.438	30364.797	126.28	185.197
29689.092	30700.776	12036.004	8236.392
32156.299	33254.086	12153.781	8416.9
35440.913	34929.878	13051.055	9494.401
38299.905	37726.606	13284.308	9659.182
38762.768	37867.838	14146.319	10117.721
62585.002	64242.746	21565.149	16585.2
63602.698	65279.001	22368.505	17875.668
64225.704	65490.793	22559.901	18098.212
12708.528	8873.513	29229.622	30254.606
12760.19	8879.114	29927.922	30853.576
12999.866	8930.018	30487.558	31281.51
13310.04	9122.808	31404.371	32319.104
13781.919	9468.931	32598.791	33575.578
20218.295	14987.103	33131.168	34065.04
20523.891	15212.43	36543.597	35715.039
20893.585	15583.633	36708.726	35955.305
39923.552	37089.877	38480.534	37286.073
39998.166	37319.777	39297.237	38007.765
40258.07	37579.066	40017.008	38285.078
41837.824	38863.123	40324.348	38688.355
42119.514	39103.472	40649.417	39007.376
42668.52	39196.482	41010.871	40056.867
43087.754	39999.654	42627.143	40408.469
44063.498	40498.051	42913.877	40757.768
44676.022	41103.746	43493.709	40997.607
46295.835	42009.414	43809.292	41398.901
47512.411	43077.896	44732.86	41821.695
47612.067	43173.678	46130.508	42900.237
48932.281	43996.109	46659.264	43283.615
51629.753	46352.42	47459.11	43859.746
51901.4	46595.667	48727.622	44862.145
52984.437	46612.306	49684.929	45357.837
60140.064	52460.275	50306.839	46173.774
63602.994	56072.051	52933.812	48229.879
70112.752	68736.591	53587.053	48589.194
71282.236	68742.871	54711.645	48779.92
72030.346	69237.737	61839.084	54998.047
72209.764	70305.098	63834.512	58602.55
73366.877	70639.859	63877.207	65415.183
74205.773	71366.018	65250.852	65863.871
76524.824	72354.426	65696.178	66627.539
76951.375	72665.565	66177.333	67420.979
77836.941	73161.034	66827.558	67613.22
		67387.951	68771.481
		71838.755	70359.637
		72823.942	70777.438
		73717.849	71279.595
		74777.371	72768.054
		75662.302	73091.616
		76253.47	74077.458
		78064.807	74465.516
		79233.216	75856.38
		…………	……………

Supplementary Table 6. CASSCF/PT2+RASSI computed spin-free and spin-orbit states for $[ReCl_4(mal)]^{2-}$ (**3**)

Spin-free Energies		Spin-Orbit Energies	
CASSCF	MS-CASPT2	CASSCF	MS-CASPT2
0	0	0	0
29317.531	27717.969	75.078	127.612
29460.773	29767.423	12005.694	7681.556
32575.41	33659.566	12522.412	8241.426
34857.677	34515.075	13348.153	9155.846
39145.527	36044.058	13489.798	9342.536
39578.776	37836.036	14316.403	9762.188
63261.096	61764.254	21783.001	16205.979
63854.152	62553.608	22415.621	17496.174
64467.233	65407.81	23022.513	18015.527
12974.742	7526.77	29092.681	30210.95
12997.115	8027.831	29850.983	30809.182
13362.309	8296.94	30313.39	31216.935
13516.484	8357.812	31421.059	32371.967
13835.592	8668.828	32980.773	33902.946
20648.381	14367.419	33483.907	34362.297
20778.442	15164.327	36100.161	35186.827
21186.624	15538.616	36295.166	35745.486
40010.81	33845.722	38819.278	36905.271
40086.336	34248.657	39728.422	37809.116
40203.161	35529.904	40329.77	38188.412
42009.29	36209.919	40479.068	38852.593
42099.994	37057.429	40816.97	39075.502
42627.999	37963.184	41756.587	39969.391
43390.38	39076.607	42837.308	40525.278
44046.54	39373.165	43129.41	40748.426
45105.697	40355.771	43558.105	41082.769
46444.863	40485.661	44170.883	41174.596
48196.091	41701.649	44668.511	41560.669
48308.617	42596.875	46135.049	42357.928
48873.746	42791.658	46808.683	42958.621
52490.311	43968.034	47639.67	43729.236
52768.638	45236.862	49219.982	44347.3
53374.954	45751.503	49635.796	44998.968
59741.63	51729.904	50799.436	46175.481
64603.036	54172.447	53532.825	48273.541
69850.698	64687.95	54330.657	48580.743
71526.315	65850.892	54889.747	49009.746
71759.277	66620.519	61290.136	53939.051
72645.68	66707.936	64020.717	58894.989
73829.832	67549.351	64443.463	65665.805
74261.517	69029.567	65241.052	65905.143
76968.829	69838.487	66155.818	66398.512
77249.322	70851.098	66451.877	67327.063
77666.507	71939.589	67181.194	67758.685
88103.97	77549.815	67725.273	68828.53
88424.908	79875.426	71211.633	69200.896
93674.485	83291.072	72954.259	70331.912
103856.845	99076.936	73541.736	70634.711
108196.728	103198.321	75007.831	72179.89
		75761.589	72681.224
		76111.056	73592.788
		77954.705	74135.459
		79372.255	75649.944
		80731.396	75996.151
		....	....

Supplementary Table 7. CASSCF+RASSI computed spin-free and spin-orbit states on the crystal structure of $[ReCl_4(cat)]^{2-}$ (**4**)

Spin-free Energies		Spin-Orbit Energies	
CASSCF	MS-CASPT2	CASSCF	MS-CASPT2
0	0	0	0
27506.862	27717.969	156.83	298.508
28878.651	29767.423	11909.687	6846.045
32549.504	33659.566	12517.69	8164.746
34655.062	34515.075	13200.46	8876.754
37813.064	36044.058	13488.83	9232.016
38468.222	37836.036	14323.888	9630.854
60867.082	61764.254	21599.567	16069.009
61899.247	62553.608	22227.3	17000.577
64083.224	65407.81	22834.968	18521.027
12805.349	7526.77	27880.061	28194.398
12889.164	8027.831	28388.681	28616.255
13205.627	8296.94	29675.153	30315.903
13472.213	8357.812	30445.491	31078.965
13934.109	8668.828	32862.941	33757.54
20395.472	14367.419	33421.665	34108.423
20639.003	15164.327	35771.852	34315.119
20968.998	15538.616	35935.962	34752.237
38308.394	33845.722	37788.759	35808.742
38437.89	34248.657	38341.529	36605.534
39522.858	35529.904	39252.036	36752.163
40435.113	36209.919	39516.665	37456.039
41210.778	37057.429	39794.408	37662.268
42149.301	37963.184	40747.958	38175.453
43149.201	39076.607	41465.341	38796.27
43505.446	39373.165	42024.935	39225.666
44479.181	40355.771	42373.549	40089.228
46405.21	40485.661	43658.613	40428.272
46583.832	41701.649	44214.236	40850.707
47449.339	42596.875	45585.656	41736.552
48287.802	42791.658	46186.577	42010.14
50885.539	43968.034	47192.682	42818.792
51574.603	45236.862	48320.223	43838.953
52519.734	45751.503	49128.903	44541.202
59449.139	51729.904	49783.54	45170.263
62873.893	54172.447	52164.791	46103.928
67533.164	64687.95	53069.312	47364.872
69566.278	65850.892	54071.184	47777.968
70192.085	66620.519	60847.899	54056.748
71009.164	66707.936	62141.245	56601.278
71734.48	67549.351	62208.237	62909.8
73450.23	69029.567	63722.748	63414.135
74407.082	69838.487	64645.701	64401.543
75469.881	70851.098	64837.713	65425.714
77219.2	71939.589	66241.046	66112.82
85997.585	77549.815	66739.117	66781.247
87818.004	79875.426	69025.265	67279.591
90689.759	83291.072	71065.318	68079.746
101546.418	99076.936	72294.374	68899.491
105708.749	103198.321	73053.238	69947.897
		73945.846	70335.321
		74915.177	71501.083
		76818.922	72860.729
		77145.064	73291.397
		79765.325	74931.285
		…………	…………

Supplementary Table 8. CASSCF/PT2+RASSI computed spin-free and spin-orbit states for $[ReCl_4(pyim)]$ (**5**)

Spin-free Energies		Spin-Orbit Energies	
CASSCF	MS-CASPT2	CASSCF	MS-CASPT2
0	0	0	0
30668.56	32429.782	22.617	72.577
31841.413	33753.172	11172.794	6782.367
32901.134	34130.107	11961.049	8666.494
37204.185	36737.027	12695.766	8860.755
38651.018	38116.964	12987.838	9215.406
39863.312	40086.621	13623.784	15478.284
64695.744	66453.648	20982.042	16305.693
66271.8	67812.817	21408.055	17825.717
66455.332	68533.042	22587.113	32535.2
12285.643	7804.712	30840.525	32944.827
12326.211	7924.208	31336.189	33895.803
12561.054	7971.495	32334.144	34135.066
12909.589	8206.091	32711.557	34447.752
12993.841	8219.024	33240.92	35038.272
19853.402	14101.107	33876.712	36653.846
20212.1	14338.398	37654.981	37196.953
20516.796	15158.885	38305.276	38326.271
40731.473	37573.102	39536.77	38708.369
41432.595	38122.571	39824.701	39248.555
42147.978	38738.99	40397.729	39588.431
42543.03	39308.692	41159.948	39794.005
43236.904	39744.109	41850.764	40889.805
43581.364	39986.458	42245.086	41079.966
44614.611	40320.513	43209.185	41495.412
44755.668	40644.349	43700.277	41775.757
46111.751	41439.783	44062.596	42259.744
46443.371	42195.515	44709.215	42652.899
47161.626	42246.629	45940.065	42920.975
48603.25	44177.485	46702.232	43705.245
49812.924	44268.786	47886.212	44154.996
51021.086	45433.639	48055.441	44604.618
52293.935	46349.264	48802.276	45672.856
53224.243	47179.372	49970.204	46131.08
61033.398	52652.321	50754.013	47205.733
62851.63	55232.74	52309.942	48176.06
72768.769	69923.653	53623.852	48894.679
73570.007	70544.705	54591.684	54968.795
74283.394	71943.633	62525.405	57486.631
74611.572	72114.251	64079.347	67627.846
74901.658	72461.7	65978.117	67750.466
76246.519	73009.144	66266.771	68833.887
77449.897	73490.708	41159.948	69584.173
79548.883	75103.792	41850.764	70040.822
79820.933	75286.528	42245.086	70943.077
89656.792	82188.157	43209.185	71750.543
89806.591	83195.57	43700.277	72266.24
91877.143	85591.644	44062.596	73417.281
107856.387	106342.99	44709.215	73916.482
109203.72	107835.411	45940.065	74771.107
		46702.232	75471.206
		47886.212	76014.744
		48055.441	77642.146
		48802.276	77958.62
		49970.204	83863.569
		....	....

Supplementary Table 9. CASSCF/PT2+RASSI computed spin-free and spin-orbit states for $[ReCl_4(bpym)]$ (**6**)

Spin-free Energies		Spin-Orbit Energies	
CASSCF	MS-CASPT2	CASSCF	MS-CASPT2
0	0	0	0
30872.402	32434.682	49.65	103.693
31273.383	32794.002	11053.268	6601.717
32707.045	33984.336	11997.37	7887.812
37155.686	36458.972	12668.269	8489.068
37650.097	36995.172	12908.898	8655.541
40094.757	40241.632	13618.469	9266.769
64032.588	65641.969	20961.619	15591.736
65719.42	67582.529	21398.266	16056.667
66448.466	67991.086	22700.302	17915.91
12286.063	7616.549	30973.626	32575.415
12311.89	7701.775	31506.243	32899.883
12419.46	7819.662	31802.874	33026.495
12817.484	7910.409	32174.379	33325.183
12907.285	8432.444	33105.329	34237.091
19738.978	13687.44	33688.499	34765.958
20452.456	14797.703	37392.565	36371.748
20500.316	15027.905	38244.086	36900.731
40491.628	37009.575	38680.043	37439.883
41530.356	38058.87	39377.084	38479.139
41879.117	38419.812	40473.4	38701.602
42494.063	39010.395	40994.34	39147.757
42687.189	39205.54	41918.216	39374.847
43511.897	39845.54	42226.718	40230.391
44371.907	40411.268	42824.846	41097.139
44538.27	40458.431	43587.379	41313.58
45679.12	40760.582	43711.534	41427.885
46142.827	41019.205	44577.429	42077.962
46356.795	41063.44	45691.778	42314.923
48745.006	43775.58	46493.045	42938.482
49610.914	44153.64	47583.877	43174.509
50047.706	44325.711	47850.928	43376.931
52406.234	45726.354	48060.108	43588.642
52894.503	47311.261	50074.508	45346.785
60823.122	52183.185	50442.944	45944.719
62649.539	54794.48	51552.848	46250.635
72207.673	69560.798	53683.536	47802.665
73313.616	70632.062	54285.697	48987.672
74129.893	71036.233	62327.425	54451.192
74382.729	71516.247	63781.942	57095.672
74740.005	71755.815	65366.989	66903.949
75220.816	72137.264	65787.752	67016.196
76751.301	72580.217	66890.692	68497.282
79173.47	74342.035	67654.02	69166.035
79332.968	74746.407	68322.526	69476.385
88293.167	80852.904	69254.088	70383.543
90251.729	83275.059	73878.311	71795.462
91026.465	84107.354	74486.478	72303.938
107702.336	105754.778	75436.987	72680.514
108123.713	106825.19	75848.226	73122.697
		76814.043	74018.32
		77219.653	74775.056
		78331.713	75140.604
		81004.54	76891.613
		81603.526	77292.294
		....	....

Supplementary Table 10. CASSCF/PT2+RASSI computed spin-free and spin-orbit states for $[ReCl_4(CN)_2]^{2-}$ (**7**)

Spin-free Energies		Spin-Orbit Energies	
CASSCF	MS-CASPT2	CASSCF	MS-CASPT2
0	0	0	0
28840.431	30778.383	42.251	34.893
34441.199	36080.698	11163.19	6570.33
34734.149	36212.491	12134.015	7924.172
42533.385	42666.444	12825.22	8823.999
43278.557	42919.075	13368.995	8991.433
44264.533	43460.154	13656.659	9603.291
71110.282	72461.306	21205.276	15737.602
71322.907	72690.238	21776.471	17229.712
73296.545	75109.227	23116.648	18804.843
12445.458	7377.315	29317.534	31180.544
12470.8	7649.243	29678.955	31540.246
12517.997	8351.557	33798.729	34962.591
12584.568	8419.678	35276.365	36199.37
13414.673	8554.35	35991.284	36294.018
20279.414	14439.948	36615.825	37848.216
20820.691	15965.569	40089.22	38715.18
20967.277	16224.169	40486.685	38810.721
39514.21	36663.936	42056.509	40024.904
40174.978	37474.969	42336.88	41514.895
40919.784	37868.906	42646.966	41842.441
43120.793	40581.18	43343.279	42222.222
43261.504	40591.798	43943.288	42483.248
44490.062	40606.555	44713.475	42643.388
46736.836	43439.376	44894.912	43620.218
47152.76	43706.958	45899.041	43720.157
50907.124	45537.746	46484.819	44716.317
51084.922	46804.5	46620.41	45224.015
51788.683	47615.522	47930.157	45775.926
54057.888	49364.98	48338.604	46105.526
55760.386	49966.586	50756.387	46786.862
55984.609	50519.308	52062.159	48075.867
56561.05	50915.067	53765.814	49280.162
56582.311	51309.833	53765.814	50850.334
62036.646	54709.461	55508.124	51541.577
67736.537	58716.896	56032.267	52013.145
72790.881	71637.659	57276.072	52404.353
72977.218	71685.676	57953.259	53314.331
78891.975	75978.339	58161.874	56828.824
78985.86	76218.608	63590.036	61022.216
81742.378	78425.358	69020.524	72441.645
82451.888	78812.126	71837.196	73103.424
83828.923	79115.463	72160.564	73445.996
83920.426	79763.876	72684.287	73863.714
87478.977	82675.736	73414.577	73998.917
94636.015	88759.238	73666.096	74802.124
99310.848	92058.363	74724.077	75899.669
100169.942	92967.642	75071.761	76502.046
113263.274	111597.707	75765.441	78641.596
121165.412	118215.995	80244.779	78846.999
		81270.233	80284.19
		83551.946	80574.433
		83685.685	81511.816
		85401.425	82156.078
		85496.764	85037.613
		....	....

Supplementary Table 11. CASSCF/PT2+RASSI computed spin-free and spin-orbit states for $[ReCl_5(py)]^-$ (**8**)

Spin-free Energies		Spin-Orbit Energies	
CASSCF	MS-CASPT2	CASSCF	MS-CASPT2
0	0	0	0
28849.947	30756.624	22.598	74.17
30614.93	32217.85	11624.828	6995.77
30883.943	32448.071	12191.673	7923.992
36609.347	35710.316	12954.719	8785.621
36649.063	36580.91	13200.301	8936.566
37108.755	37212.296	13891.865	9369.966
62107.006	64261.387	21224.802	15595.878
62598.614	64640.892	21767.851	16647.925
63334.616	65656.392	22554.693	17778.648
12616.34	7841.718	29114.723	30977.885
12690.495	8089.278	29621.762	31393.51
13054.093	8233.677	31018.303	32274.045
13110.143	8290.974	31293.043	32600.952
13350.608	8419.232	31518.781	32861.487
20254.766	14316.893	32010.05	33463.505
20317.055	14536.46	36205.537	35384.856
20581.982	14989.662	37359.694	35922.981
39391.011	36041.65	37750.885	36695.649
39420.442	36944.51	38111.158	36942.013
40683.593	37650.505	38483.334	38178.883
41135.573	38316.242	38980.625	38431.091
41369.539	38481.355	40191.856	38681.354
42345.946	38898.497	40304.268	39060.444
42684.712	39014.744	41811.745	39462.063
42797.651	39485.68	42071.576	39798.242
44768.254	40451.719	42229.929	40217.734
45202.329	41255.638	43290.226	40841.344
45624.464	41720.773	44372.853	41538.977
45979.983	41786.647	44901.376	41821.843
49264.433	43664.334	46521.783	42712.577
49478.779	44490.703	46909.015	43000.983
49871.426	45018.473	46968.867	43529.462
51548.801	45050.128	47635.791	44324.545
60243.897	52757.087	49723.254	45105.981
60642.219	52898.898	51010.028	46220.18
69908.37	67106.187	51421.587	46851.549
70214.904	67981.535	52951.135	46963.589
70587.396	68628.284	61433.546	55055.332
70906.341	68952.568	61754.7	55177.171
71421.715	69905.998	63224.126	65226.141
73120.247	71098.688	63715.094	65459.284
74917.021	71220.236	64048.594	65874.058
75175.888	71459.367	64950.116	66764.471
76571.56	73169.852	65384.894	67324.779
86228.143	80062.734	66399.889	68187.851
86870.964	80458.258	71056.772	68796.815
87369.778	81078.775	71777.861	69464.739
102977.089	102377.099	71950.992	70681.53
104407.913	103664.406	72729.539	71144.445
		74120.318	72592.499
		74551.269	72917.025
		75973.986	73203.665
		77578.025	74626.386
		78864.449	75778.139
		…………	…………

Supplementary Table 12. CASSCF/PT2+RASSI computed spin-free and spin-orbit states for [$ReCl_4(py)_2$] (**9**)

Spin-free Energies		Spin-Orbit Energies	
CASSCF	CASPT2	CASSCF	MS-CASPT2
0	0	0	0
30580.269	32186.229	21.809	116.627
33870.271	35031.55	10635.239	5530.665
34360.108	35193.995	12206.104	7509.801
38082.924	36551.654	12814.926	8691.57
40398.021	39934.038	13519.352	9160.968
40910.972	40458.26	13682.52	9424.388
66584.741	68626.835	21633.859	15912.626
67886.119	69330.016	22013.828	17573.599
69943.946	72651.582	23856.773	19766.571
11783.492	5950.56	30978.099	32640.72
12479.151	6683.809	31384.836	33064.742
12517.315	8137.028	34148.313	35034.883
12676.998	8399.434	34415.098	35168.398
13587.355	8499.843	34713.395	35316.096
20800.899	14464.658	35276.996	36057.536
20882.852	16204.16	38519.017	37124.333
21812.633	17208.273	39257.238	37741.697
41053.213	37779.017	40795.902	38702.85
41390.81	38174.343	41142.131	39429.089
42133.282	39162.413	41606.903	40249.913
44525.961	40095.031	42059.898	40855.267
44798.135	41282.463	42557.489	41393.174
44881.839	41338.7	42969.231	41896.131
45728.434	41665.233	44017.379	42083.408
46462.813	42418.282	45199.559	42716.236
46968.77	42519.598	45273.662	42864.095
49168.488	44065.809	46102.727	43483.742
49358.736	44694.804	47342.257	43747.458
49626.739	44759.924	47805.833	44535.826
50916.461	46230.499	49100.514	44902.728
53302.926	47091.317	50441.214	46110.12
53449.493	47861.094	50839.915	46307.164
54348.176	47985.363	51171.304	46945.559
63210.129	54339.203	52080.834	48283.791
64428.666	58502.642	54165.67	49106.326
74868.181	72102.845	55037.216	49517.699
74988.912	72744.04	55735.349	50049.641
75522.48	73024.051	64701.893	56942.24
76325.449	73522.793	65705.087	60787.012
78149.079	75126.178	67843.987	69878.894
80254.827	75292.395	68194.876	70264.459
80286.46	76994.446	69281.087	70960.462
80461.837	77838.099	70008.694	71845.861
83543.314	80569.472	71578.435	73265.374
92358.691		72353.223	73677.968
93220.573		76012.635	74622.104
93301.557		76760.452	74839.933
111053.222		77090.669	76006.415
113084.592		78149.017	76254.716
		80394.536	77169.625
		80960.344	78233.815
		81427.49	79658.353
		83421.961	80686.503
		85486.006	83031.482
		....	....

Supplementary Table 13. CASSCF/PT2+RASSI computed spin-free and spin-orbit states for $[ReCl_5(pyd)]^-$ (**10**)

Spin-free Energies		Spin-Orbit Energies	
CASSCF	MS-CASPT2	CASSCF	MS-CASPT2
0	0	0	0
29463.475	31375.761	16.359	88.929
31276.748	32910.382	11309.944	6578.683
31591.556	33038.322	11973.69	7531.868
36814.068	36083.543	12737.693	8442.208
37528.814	37364.828	13061.994	8619.136
37805.476	37696.319	13682.363	9134.501
63184.853	65180.967	20916.241	15118.687
63642.457	65313.503	21531.271	16548.591
64719.29	66919.603	22518.768	17696.448
12381.382	7385.915	29731.441	31625.965
12480.781	7601.111	30205.474	32016.901
12719.532	7686.918	31594.738	32893.836
12791.707	7946.953	31959.234	33281.349
13134.063	8196.639	32115.157	33341.516
19906.808	13651.654	32670.618	34007.417
20215.045	14807.986	36706.317	35597.97
20510.886	14881.851	37768.617	36400.295
39801.95	36361.317	38413.386	37142.975
39854.96	36706.573	38622.954	37286.689
41031.209	37693.041	39258.536	38430.771
41813.607	38476.395	39349.74	38894.883
42062.561	38909.276	40694.087	39090.323
42902.133	39070.315	40862.3	39478.12
42962.789	39458.602	42244.933	39857.203
43221.092	39610.662	42549.139	40181.49
45335.52	40109.683	42711.722	40425.339
45481.351	41758.734	43812.181	40959.109
46326.203	42021.058	44822.892	41570.683
46557.915	42252.609	45239.545	42207.043
49180.145	43097.298	47000.837	42560.014
50104.631	44910.125	47319.125	43590.205
50282.46	45095.973	47607.663	43897.428
51889.356	45209.4	48143.482	44521.098
60474.999	51945.144	49948.12	44850.682
60991.357	53903.486	51284.796	46429.933
71059.308	68021.852	51864.716	46939.173
71290.21	68530.069	53268.665	47137.615
71674.52	69467.854	61834.153	54332.743
71985.34	69902.423	62226.676	56139.916
72754.908	70877.113	64248.177	66022.527
74253.481	71399.611	64645.164	66336.382
75869.854	71628.861	65023.653	66710.811
76142.871	71900.913	65923.836	67569.63
77825.475	74003.501	66618.827	68370.672
87580.03	79193.712	67580.252	68993.972
87730.874	79680.693	72198.492	69904.585
88049.861	82425.217	72845.165	70371.607
104739.015	103763.701	73094.343	71544.15
105871.518	104538.723	73733.182	71789.112
		75296.668	73111.411
		75584.661	73490.992
		76936.346	73956.209
		78598.76	74839.912
		79983.108	76318.208
		…………	…………

Supplementary Table 14. CASSCF/PT2+RASSI computed spin-free and spin-orbit states for $[ReCl_5(pym)]^-$ (**11**)

Spin-free Energies		Spin-Orbit Energies	
CASSCF	CASPT2	CASSCF	MS-CASPT2
0	0	0	0
29168.291	31282.448	12.359	51.589
30207.814	31528.681	11598.467	7368.57
30437.125	31594.281	12083.652	7990.062
36110.082	35828.971	12891.669	8932.625
36653.676	36439.923	13108.241	8980.834
36843.728	36549.438	13826.085	9464.255
61720.72	64010.147	21084.662	15647.603
62214.362	64174.819	21687.588	16869.344
62745.759	65072.415	22342.807	17561.396
12516.187	8300.748	29318.098	31115.436
12608.799	8332.961	29859.719	31584.102
12999.203	8341.608	30539.762	31639.828
13125.03	8501.302	30979.274	32110.471
13269.137	8549.611	31086.925	32262.401
20127.915	14353.803	31677.926	32909.703
20212.462	14730.499	35967.315	35046.837
20356.289	14779.576	37085.185	36172.729
39426.975	36487.378	37543.618	36412.196
39642.678	36982.373	37852.719	36790.613
40786.826	37698.587	38429.309	38084.214
40920.356	37870.288	38529.635	38141.251
41008.763	38006.579	40270.019	38382.602
41931.075	38753.26	40407.179	38707.282
42117.843	38893.855	41373.931	39109.909
42675.298	38947.805	41592.074	39373.157
44095.052	40278.489	42194.601	39872.016
44829	40941.486	43170.378	40746.076
45508.059	41029.638	43899.267	41063.851
45618.403	41092.04	44762.924	41575.289
48708.59	43314.294	45957.749	42355.659
49364.997	44269.584	46519.793	42949.065
49479.626	44352.001	46873.261	42996.649
51280.331	44913.917	47217.155	43495.562
59944.289	51930.577	49340.099	44686.47
60233.777	53161.073	50656.442	45899.242
69775.123	67251.734	51088.489	46323.901
69968.904	67691.724	52671.682	46659.996
70417.322	68244.558	61145.275	54243.64
70614.298	68513.501	61293.099	55368.531
70797.931	69315.055	62813.098	64848.147
72031.663	70168.124	63362.352	65069.898
74621.588	70460.795	63571.708	65339.402
74694.806	70617.662	64513.293	66304.159
75864.479	72550.025	64864.541	66736.429
86122.11	79149.75	65901.143	67719.021
86214.637	79335.596	70935.01	68806.294
86492.084	80367.613	71513.965	69264.572
102622.199	102036.941	71658.443	70176.847
103318.689	102554.927	72224.478	70441.161
		73600.304	71764.965
		73733.017	72175.195
		75447.342	72506.35
		77242.701	74043.351
		78138.27	74881.785
		…………	…………

Supplementary Table 15. CASSCF/PT2+RASSI computed spin-free and spin-orbit states for $[ReCl_5(pyz)]^-$ (**12**)

Spin-free Energies		Spin-Orbit Energies	
CASSCF	CASPT2	CASSCF	MS-CASPT2
0	0	0	0
29309.276	31121.444	17.932	68.872
30185.711	31780.309	11494.184	6801.578
30719.131	32207.616	12053.713	7630.613
35778.018	35152.913	12837.532	8539.826
36975.631	36828.255	13133.422	8631.678
37221.423	36944.337	13793.946	9129.715
61960.376	63825.345	21058.136	15265.417
62247.475	64059.607	21645.982	16544.289
63196.424	65319.508	22472.881	17520.213
12507.538	7630.29	29463.932	31209.259
12585.757	7813.723	30009.189	31646.515
12904.738	7929.666	30559.098	31898.48
12963.06	8049.536	31058.823	32366.214
13253.788	8119.458	31251.214	32515.99
20081.767	13853.921	31879.541	33222.134
20267.268	14592.583	36007.225	34912.247
20463.549	14693.69	36881.612	35821.335
39491.335	36149.198	37783.571	36522.591
39705.196	36579.878	38132.551	36740.623
40861.569	37329.814	38332.649	37773.26
40930.196	37836.652	38936.416	38109.566
41386.138	38216.046	40352.202	38521.915
41972.39	38455.484	40627.685	38909.887
42332.961	38651.773	41373.738	39247.487
42666.492	38947.859	41754.118	39551.405
44315.562	39637.882	42309.085	39768.429
44625.862	40791.372	43331.554	40583.326
45782.291	40959.617	44023.109	41059.35
45840.422	41047.687	44790.624	41286.473
48405.312	42539.882	46153.522	41945.624
49618.919	44380.579	46379.24	42797.067
49778.013	44560.623	47202.265	43043.282
51338.364	44664.687	47366.136	43463.373
59682.364	51340.261	49243.455	44194.528
60733.638	53464.543	50750.431	45919.276
69946.235	67630.597	51351.648	46464.058
70189.215	67846.578	52731.767	46520.722
70441.873	68577.445	60986.234	53724.82
71072.511	69024.703	61681.687	55689.765
71203.986	69418.355	63016.877	64734.894
72223.4	70225.036	63601.246	65005.645
74687.956	70574.952	63760.929	65370.504
74969.403	70856.88	64653.861	66261.894
76196.81	72562.73	65212.896	66908.862
86091.602	79344.124	66190.111	67761.333
86398.985	79676.728	71174.206	69469.548
87002.144	81033.734	71583.169	69844.122
103078.018	102142.202	72104.88	70343.7
103550.07	102473.487	72425.397	70637.049
		73848.673	71901.012
		73983.632	72147.391
		75604.239	72769.149
		77495.943	74197.968
		78397.298	75003.144
		....	....

Supplementary Table 16. CASSCF/PT2+RASSI computed spin-free and spin-orbit states for $[ReCl_5(dmf)]^-$ (**12**)

Spin-free Energies		Spin-Orbit Energies	
CASSCF	MS-CASPT2	CASSCF	MS-CASPT2
0	0	0	0
28723.106	30442.397	14.24	38.876
29160.678	30594.088	12002.621	7807.245
29307.003	30851.548	12166.088	8120.64
35581.461	35263.36	13144.87	9108.158
35727.283	35431.761	13215.925	9318.03
35871.972	35515.356	14055.198	9627.826
60412.582	62170.218	21351.502	15959.892
60543.392	62682.518	22075.889	17263.379
60734.563	63002.447	22269.522	17524.121
12798.657	8467.423	28684.648	30290.568
12799.872	8699.561	29325.302	30791.572
13374.167	8784.809	29647.37	30849.221
13394.593	8822.949	30054.054	31325.344
13494.035	8849.486	30164.041	31416.75
20347.827	14701.343	30736.362	32125.019
20408.294	14728.964	35263.995	34283.167
20458.905	14828.129	36542.817	35550.224
38944.776	35788.223	36624.344	35613.548
39320.577	36750.074	37174.967	36315.355
40041.457	36981.666	37542.538	37446.643
40119.772	37288.85	37909.777	37561.448
40646.529	37817.679	39658.729	37616.802
41102.605	37862.369	39939.85	38101.87
41220.861	38106.317	40636.298	38335.751
42405.036	38694.128	40883.111	38771.864
42845.616	39633.173	41719.375	39228.399
44608.646	40037.706	42659.799	40249.87
44662.202	40051.646	43109.291	40365.043
44880.834	40238.807	44474.619	41264.6
48588.27	43363.107	44878.01	41798.845
48663.545	43619.944	45987.808	42154.241
48888.17	43641.871	46261.343	42241.036
50759.628	44391.765	46617.173	42730.919
59316.798	51982.252	48897.946	44609.514
60007.452	52116.833	50339.846	45582.282
68531.234	65920.54	50473.856	45705.92
68573.909	66019.433	52199.402	46409.187
68761.913	66910.071	60195.575	54462.148
69154.097	67105.681	60569.034	54565.649
69302.413	67424.397	61373.734	63260.816
70061.703	68036.999	62144.467	63572.863
73402.658	69450.61	62400.457	63892.031
73466.125	69496.49	63126.868	64733.977
73993.152	70504.073	63286.44	65009.344
84347.016		64273.205	66084.182
85188.952		69572.28	67874.577
85381.581		70051.415	68051.836
100278.746		70322.151	68682.273
101036.68		70764.499	69250.978
		72051.45	70519.141
		72180.513	70784.442
		74122.142	70948.848
		75979.454	73055.619
		76543.241	73470.234
		…………	…………

Supplementary Table 17. MS-CASPT2 +RASSI/SINGLE_ANISO computed D, E, and g-values for complex **1-13**. The values given in the parenthesis are the computed values at CASSCF level.

Complex	D_{tot}	|E/D|	g_{xx}	g_{yy}	g_{zz}
$[ReBr_4(ox)]^{2-}$	-93.02	0.18	1.7261	1.7126	1.6983
	(-63.99)	(0.21)	(1.7742)	(1.7814)	(1.7916)
$[ReCl_4(ox)]^{2-}$	-85.04	0.24	1.7358	1.7266	1.6988
	(-57.51)	(0.26)	(1.7858)	(1.7806)	(1.7584)
$[ReCl_4(mal)]^{2-}$	-61.67	0.15	1.7225	1.7157	1.6907
	(-35.60)	(0.19)	(1.7821)	(1.7794)	(1.7591)
$[ReCl_4(cat)]^{2-}$	±132.65	0.30	1.7248	1.7088	1.6446
	(±70.44)	(0.28)	(1.7904)	(1.7849)	(1.7448)
$[ReCl_4(bpmn)]$	-47.91	0.23	1.7299	1.7191	1.7043
	(24.79)	(0.02)	(1.7995)	(1.7969)	(1.7873)
$[ReCl_4(pyim)]$	-34.73	0.17	1.7289	1.7222	1.7091
	(±9.42)	(0.38)	(1.7882)	(1.7906)	(1.7966)
$[ReCl_4(CN)_2]^{2-}$	16.19	0.23	1.7565	1.7519	1.7299
	(-20.97)	(0.06)	(1.8279)	(1.8251)	(1.7888)
$[ReCl_5(py)]^{-}$	±32.65	0.31	1.7190	1.7142	1.7064
	(-10.36)	(0.25)	(1.7870)	(1.7856)	(1.7809)
$[ReCl_4(py)_2]^{-}$	55.65	0.18	1.6831	1.6870	1.7016
	(-10.78)	(0.08)	(1.8000)	(1.7954)	(1.7906)
$[ReCl_5(pyd)]^{-}$	41.00	0.24	1.7219	1.7120	1.699
	(7.92)	(0.14)	(1.7922)	(1.7914)	(1.7862)
$[ReCl_5(pym)]^{-}$	24.64	0.17	1.7174	1.720	1.729
	(±5.42)	(0.31)	(1.7830)	(1.7845)	(1.7864)
$[ReCl_5(pyz)]^{-}$	32.53	0.20	1.7009	1.7085	1.7198
	(8.95)	(0.03)	(1.7832)	(1.7844)	(1.7892)
$[ReCl_4(dmf)]^{-}$	18.51	0.18	1.7046	1.7073	1.7274
	(6.74)	(0.19)	(1.7625)	(1.7702)	(1.7818)

Supplementary Table 18. MS-CASPT2+RASSI/SINGLE_ANISO computed g-values for complex **1-13** assuming pseudo spin ½.

Complex	g_{xx}	g_{yy}	g_{zz}
$[ReBr_4(ox)]^{2-}$	0.7985	1.0260	4.9171
$[ReCl_4(ox)]^{2-}$	1.0029	1.3283	4.8084
$[ReCl_4(mal)]^{2-}$	0.6812	0.8461	4.9483
$[ReCl_4(cat)]^{2-}$	1.2086	1.7620	4.3864
$[ReCl_4(bpmn)]$	1.0500	1.2082	4.8477
$[ReCl_4(pyim)]$	0.7583	0.8892	4.9861
$[ReCl_4(CN)_2]^{2-}$	1.4117	2.2124	4.4856
$[ReCl_5(py)]^{-}$	1.1572	1.6059	4.6953
$[ReCl_4(py)_2]^{-}$	1.3165	2.337	4.1141
$[ReCl_5(pyd)]^{-}$	1.3637	2.137	4.381
$[ReCl_5(pym)]^{-}$	1.5002	2.4625	4.2473
$[ReCl_5(pyz)]^{-}$	1.4295	2.3492	4.2697
$[ReCl_4(dmf)]^{-}$	1.5594	2.4659	4.2030

Supplementary Table 19. NEVPT2 computed D, E and *g* values for complex **1** along with individual contribution to the D-tensor. All these calculations have been performed using ORCA 3.0 code.

Complex **1**			Effective Hamiltonian Approach		2nd Order Perturbation Approach	
States		Excited states	Contribution to D (cm^{-1})	Contribution to E (cm^{-1})	Contribution to D (cm^{-1})	Contribution to E (cm^{-1})
$^4T_{2g}(F)$		28946.5	-125.950	-0.319	-126.458	-0.594
		29404.6	54.530	54.553	55.019	55.046
		32504.0	61.507	-62.334	61.198	-62.633
$^4T_{1g}(F)$		32647.2	0.077	-0.066	0.078	-0.066
		37495.7	-7.056	0.012	-7.024	-0.003
		37605.7	2.996	3.024	3.000	3.027
^2G	^2E_g	11208.2	-0.016	-0.026	-0.016	-0.026
		11212.3	0.005	0.001	0.005	0.001
	$^2T_{1g}$	11262.9	-1.803	-1.803	-1.804	0.711
		10898.9	0.007	-0.002	0.007	-0.002
		11577.6	-2.827	0.041	2.790	0.054
	$^2T_{2g}$	17304.1	-134.184	-134.188	-138.321	-138.313
		17478.4	-112.630	112.615	-115.386	115.445
		17669.2	181.447	-0.012	184.914	0.051
Other doublet excited state		18156.8	73.843	0.207	73.657	0.365
		37623.3	-27.131	-27.078	-27.209	-27.153
		41050.0	-36.224	36.701	-35.829	36.657
			Overall D = – 85.13	Overall E/D = – 0.21	Overall D = -71.74	Overall E/D = 0.30
			g-values obtained from Effective Hamiltonian Approach		g-values obtained from 2nd Order Perturbation Approach	
			1.711792, 1.738738, 1.747494		0.941166, 1.223346, 4.898340	

Supplementary Table 20. NEVPT2 computed D, E and g values for complex **7** along with individual contribution to the D-tensor. All the calculations have been performed using ORCA 3.0 code.

<table>
<tr><td colspan="2">Complex 7</td><td></td><td colspan="2">Effective Hamiltonian Approach</td><td colspan="2">2nd Order Perturbation Approach</td></tr>
<tr><td colspan="2"></td><td></td><td></td><td></td><td></td><td></td></tr>
<tr><td colspan="2">States</td><td>Excited states</td><td>Contribution to D (cm^{-1})</td><td>Contribution to E (cm^{-1})</td><td>Contribution to D (cm^{-1})</td><td>Contribution to E (cm^{-1})</td></tr>
<tr><td colspan="2">$^4T_{2g}$</td><td>30708.5
36395.0
36595.8</td><td>2.008
25.422
-23.289</td><td>42.781
-24.743
1.579</td><td>-118.753
34.204
25.964</td><td>2.740
0.694
-2.035</td></tr>
<tr><td colspan="2">$^4T_{1g}$</td><td>42665.2
43088.6
43819.8</td><td>-0.020
-13.741
11.065</td><td>-0.001
0.915
-10.941</td><td>-0.030
15.593
15.202</td><td>0.014
-1.437
0.570</td></tr>
<tr><td rowspan="3">^2G</td><td>^2E_g</td><td>10160.7
10197.8</td><td>-8.591
8.655</td><td>8.450
-1.439</td><td>-10.820
-7.886</td><td>0.890
-1.482</td></tr>
<tr><td>$^2T_{1g}$</td><td>10626.4
10898.9
11374.4</td><td>2.043
-0.152
-0.072</td><td>0.151
0.202
-0.071</td><td>-2.718
-0.232
0.239</td><td>0.711
0.239
0.002</td></tr>
<tr><td>$^2T_{2g}$</td><td>17304.1
18477.7
18681.7</td><td>6.633
-59.176
55.361</td><td>-65.796
61.690
-3.822</td><td>185.633
-89.485
-70.229</td><td>-4.300
-8.007
10.451</td></tr>
<tr><td colspan="2">Other doublet excited state</td><td>39311.6
43918.8
44017.1</td><td>-1.336
-14.082
12.663</td><td>-27.026
13.623
-0.849</td><td>74.592
-18.818
-14.146</td><td>-1.738
-0.391
1.160</td></tr>
<tr><td colspan="2"></td><td></td><td>Overall
D = 6.767</td><td>Overall
E/D = 0.19</td><td>Overall
D = -13.47</td><td>Overall
E/D = 0.08</td></tr>
<tr><td colspan="2"></td><td></td><td colspan="2">g-values obtained from Effective Hamiltonian Approach</td><td colspan="2">g-values obtained from 2nd Order Perturbation Approach</td></tr>
<tr><td colspan="2"></td><td></td><td colspan="2">1.748624, 1.778213, 1.781092</td><td colspan="2">1.601613, 2.491766, 4.380983</td></tr>
</table>

Supplementary Table 21.CASSCF computed D, g, and E values for model complex $[ReCl_6]^{2-}$ which mimic the complex **1**. All the D and E values are provided in the cm^{-1}.

Energies of KDs (cm^{-1})	$[ReCl_6]^{2-}$
0.0<br>92.6	Xa 0.847059 -0.522850 -0.095497<br>Ya 0.450371 0.610670 0.651343<br>Za -0.282237 -0.594735 0.752750
g_X = 1.8095<br>g_Y = 1.8322<br>g_Z = 1.8550	D = -45.1036<br>E = 6.0884<br>Za is the orientation of the main magnetic axes

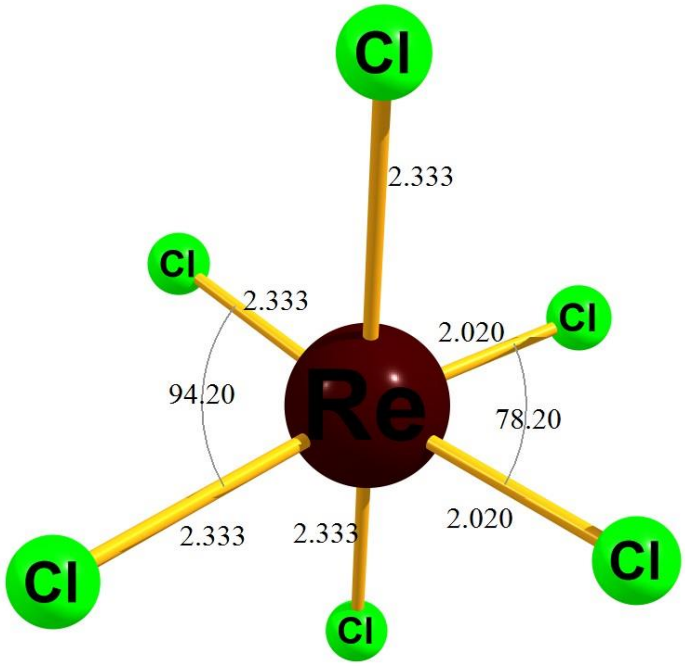

In the model complexes, structural parameters are adjusted to mimic the structural parameter of complex **1**. The –*cis* ∠Cl–Re–Cl bond angle (~ 78 degree) and –cis Re–Cl bond lengths (2.0Å) are adjusted to represent the binding mode –oxalate ligand.

Supplementary Table 22. DFT computed hyperfine splitting of ^{186}Re, ^{35}Cl and ^{80}Br in complex **1** and **2**. All the values are provided in the MHz.

**Complexes**	**Calculated |A|**	**Exp |A|**
$[ReCl_4(ox)_2]^{2-}$		
A^{Re}_x	-1961.6	1079.2
A^{FC}	-1734.9	
A^{SD}_x	1.7	
A^{SOC}_x	-228.5	
A^{Re}_y	-1966.8	1349.2
A^{FC}	-1734.9	
A^{SD}_y	1.4	
A^{SOC}_y	-233.4	
A^{Re}_z	-1968.9	1229.1
A^{FC}	-1734.9	
A^{SD}_z	-3.1	
A^{SOC}_z	-230.9	
Cl_1 $(A_{iso}{}^{FC})$	1.79	
Cl_2 $(A_{iso}{}^{FC})$	2.66	
Cl_3 $(A_{iso}{}^{FC})$	2.54	
Cl_4 $(A_{iso}{}^{FC})$	1.91	
$[ReBr_4(ox)_2]^{2-}$		
A^{Re}_x	-1661.2	1229.1
A^{FC}	-1450.2	
A^{SD}_x	-12.4	
A^{SOC}_x	198.5	
A^{Re}_y	1667.9	1229.1
A^{FC}	-1450.2	
A^{SD}_y	4.1	
A^{SOC}_y	-221.9	
A^{Re}_z	-1668.9	1229.1
A^{FC}	-1450.2	
A^{SD}_z	8.4	
A^{SOC}_z	-227.2	
Br_1 $(A_{iso}{}^{FC})$	8.05	
Br_2 $(A_{iso}{}^{FC})$	7.83	
Br_3 $(A_{iso}{}^{FC})$	8.95	
Br_4 $(A_{iso}{}^{FC})$	8.87	

Supplementary Table 23. DFT optimized geometry (PBE functional; Lanl08d for Re and TZV for rest) of the model complexes $[ReCl_4(C_6H_4E_2)]^{2-}$ where (E =S, Se). Ab initio computed orientation of main magnetic axis (Z_A) representing the D_{ZZ} axis

DFT optimized geometry of the $[ReCl_4(C_6H_4S_2)]^{2-}$			
Re	-0.879325000	-0.000001000	0.000004000
Cl	-2.479157312	1.855541122	0.000070397
Cl	-2.479178249	-1.855525071	0.000070397
Cl	-0.950325883	-0.000001976	-2.448966983
Cl	-0.950101389	-0.000006858	2.448981481
C	2.505055000	0.704620000	-0.000036000
C	2.505056000	-0.704622000	-0.000041000
C	3.733796000	-1.389709000	-0.000015000
H	3.722135000	-2.476266000	-0.000015000
C	4.950293000	-0.701076000	0.000019000
H	5.887762000	-1.253685000	0.000041000
C	4.950292000	0.701077000	0.000003000
H	5.887761000	1.253686000	0.000019000
C	3.733794000	1.389709000	-0.000008000
H	3.722133000	2.476265000	-0.000004000
S	0.950959000	1.689733000	-0.000076000
S	0.950962000	-1.689736000	-0.000084000

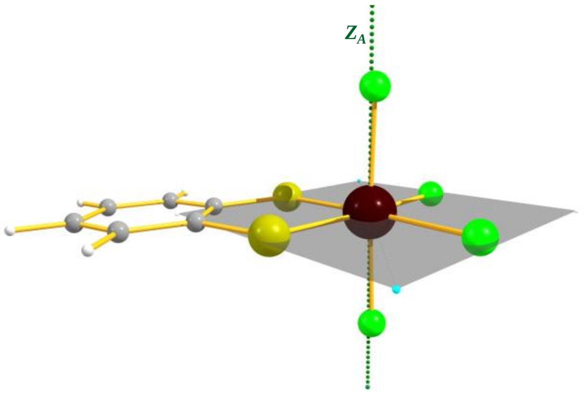

DFT optimized geometry of the $[ReCl_4(C_6H_4Se_2)]^{2-}$			
Re	-1.029300000	0.000000000	0.000010000
Cl	-2.637316000	1.843737000	0.000039000
Cl	-2.637318000	-1.843735000	0.000038000
Cl	-1.129868000	0.000001000	-2.433891000
Cl	-1.129772000	-0.000002000	2.433913000
C	2.420571000	0.703866000	-0.000018000
C	2.420571000	-0.703866000	-0.000019000
C	3.648019000	-1.381072000	-0.000016000
H	3.639002000	-2.466231000	-0.000015000
C	4.859624000	-0.697897000	-0.000013000
H	5.794105000	-1.250868000	-0.000011000
C	4.859624000	0.697897000	-0.000012000
H	5.794105000	1.250868000	-0.000008000
C	3.648019000	1.381072000	-0.000014000
H	3.639002000	2.466231000	-0.000012000
Se	0.812873000	1.732533000	-0.000026000
Se	0.812873000	-1.732534000	-0.000028000

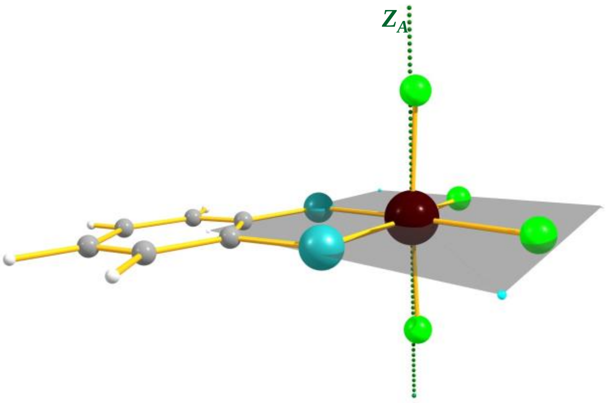

Supplementary Table 24. CASSCF and MS-CASPT2 computed D, g, and E values for model complexes $[ReCl_4(C_6H_4E_2)]^{2-}$ where (E =S, Se). All the D and E values are provided in the cm^{-1}.

<table>
<tr><td></td><td colspan="2">Spin-free energy</td><td>Computed D values@CASSCF</td></tr>
<tr><td rowspan="2">Energies of KDs (cm^{-1})</td><td>$^4A_2$</td><td>0.000</td><td rowspan="2">$[ReCl_4(C_6H_4S_2)]^{2-}$ Complex **4a**</td></tr>
<tr><td>^2E</td><td>12290.446
12431.818</td></tr>
<tr><td>0.0
66.1</td><td>$^2T_1$</td><td>12624.102
12638.981
13339.855</td><td>Xa 1.000000 -0.000005 0.000012
Ya 0.000005 1.000000 0.000413
Za -0.000012 -0.000413 1.000000</td></tr>
<tr><td>g_X = 1.7057
g_Y = 1.7200
g_Z = 1.7612</td><td>$^2T_2$</td><td>20201.291
20906.871
21388.222</td><td>D = -32.7523
|E| = 2.6441
Za is the orientation of the main magnetic axes</td></tr>
<tr><td></td><td colspan="2">Spin-free energy</td><td>Computed D values@MS-CASPT2</td></tr>
<tr><td rowspan="2">Energies of KDs (cm^{-1})</td><td>$^4A_2$</td><td>0.000</td><td rowspan="2">$[ReCl_4(C_6H_4S_2)]^{2-}$ Complex **4a**</td></tr>
<tr><td>^2E</td><td>5613.287
6907.971</td></tr>
<tr><td>0.0
66.1</td><td>$^2T_1$</td><td>7515.267
8091.662
8489.868</td><td>Xa 0.999980 0.006309 0.000005
Ya -0.006309 0.999980 -0.000029
Za -0.000005 0.000029 1.000000</td></tr>
<tr><td>g_X = 1.6440
g_Y = 1.6477
g_Z = 1.6784</td><td>$^2T_2$</td><td>14653.634
16015.501
17862.034</td><td>D = +111.8772
|E| = -25.8535
Za is the orientation of the main magnetic axes</td></tr>
<tr><td></td><td colspan="2">Spin-free energy</td><td>Computed D values@CASSCF</td></tr>
<tr><td rowspan="2">Energies of KDs (cm^{-1})</td><td>$^4A_2$</td><td>0.000</td><td rowspan="2">$[ReCl_4(C_6H_4Se_2)]^{2-}$ Complex **4b**</td></tr>
<tr><td>^2E</td><td>12232.820
12414.291</td></tr>
<tr><td>0.0
241.012</td><td>$^2T_1$</td><td>12555.095
12564.786
13264.874</td><td>Xa 1.000000 -0.000031 -0.000002
Ya 0.000031 1.000000 -0.000169
Za 0.000002 0.000169 1.000000</td></tr>
<tr><td>g_X = 1.7120
g_Y = 1.7206
g_Z = 1.7650</td><td>$^2T_2$</td><td>20032.916
20825.123
21167.254</td><td>D = -29.1640
|E| = 0.2656
Za is the orientation of the main magnetic axes</td></tr>
<tr><td></td><td colspan="2">Spin-free energy</td><td>Computed D values@MS-CASPT2</td></tr>
<tr><td rowspan="2">Energies of KDs (cm^{-1})</td><td>$^4A_2$</td><td>0.000</td><td rowspan="2">$[ReCl_4(C_6H_4Se_2)]^{2-}$ Complex **4b**</td></tr>
<tr><td>^2E</td><td>5299.630
6704.989</td></tr>
<tr><td>0.0
248.44</td><td>$^2T_1$</td><td>7354.223
7964.690
8368.765</td><td>Xa 0.999992 -0.003917 0.000078
Ya 0.003917 0.999992 0.000057
Za -0.000078 -0.000056 1.000000</td></tr>
<tr><td>g_X = 1.6388
g_Y = 1.6500
g_Z = 1.6777</td><td>$^2T_2$</td><td>14454.443
15917.206
17709.098</td><td>D = + 114.3603
|E| = 28.0062
Za is the orientation of the main magnetic axes</td></tr>
</table>

Supplementary Table 25. DFT analysed NBO representing the nature of Re-E bonds (E=O, S and Se) in complexes **4**, **4a** and **4b**.

	Complex **4**	Complex **4a**	Complex **4b**
NBO Analysis	Re–O	Re–S	Re–Se
	Re(dxy): 18.4<br>O(p_x): 81.6%	Re(dxy): 32.4<br>O(p_x): 67.6%	Re(dxy): 36.6<br>O(p_x): 63.4%

Supplementary note 1. Nature of Excitations governing zfs. For an ideal octahedral ligand field environment around Re(IV) ion, D must be zero as all these mentioned excitations will cancel each other due to cubic symmetry. Any deviation from the ideal O_h geometry led to significant mixing between ground and excited states which is the origin of D values. The mixing of $^4A_{2g}$ with $^4T_{2g}$ is a symmetry allowed transition in O_h environment and these three excitations are usually the main contributor to the D values from the spin-conserved excitations, where only electron gets excited. However in $^4A_{2g}\rightarrow^4T_{1g}$(F) excitations, two electrons gets promoted from the t_{2g} sub-shell to e_g sub-shells at a time. In spin-flip excitations, ^2G ($^2T_{2g}$, $^2T_{1g}$ and ^2E) states are rigorously mixed with $^4A_{2g}$ ground state as they are low-lying compared to other quartet states. Our calculation suggests that $^2T_{2g}$ ground state contribute significantly to the D values. The three excitations (υ_1-υ_3) are the major contributor to the D values. In low-symmetry environment, it is difficult to generalize the major contribution as all these states mixed strongly with the ground state.

Supplementary note 2 – Residual error plots for magnetisation data. In order to compute the residual error for the fitting the magnetisation data using D and E values we have used the survey feature using the PHI code by varying D and E values by fixing the g_{xx}, g_{yy} and g_{zz} values. The residual error is the product of the residuals errors of the magnetisation and susceptibility curves simulated together with the chosen parameters compared with the experimental data. Here survey has been performed on complex **1** and **7** by varying D from -100 cm^{-1} to +100 cm^{-1} and E values from -30 cm^{-1} to +30 cm^{-1} by fixing g values.

Supplementary note 3 – Ab initio blockade barrier for magnetisation reversal. *Insights from ab initio blockade barrier:* Here we have computed the transversal magnetic moment between the connecting pairs to analyse the mechanism of magnetic relaxation for complex **1**. The thick black line indicates the Kramers doublets (KDs), as a function of magnetic moment. The grey colour lines shown in figures which are connected to the ground state KD are the pictorial representation of the hyperfine splitting of the ground state KD. The dotted green lines show the possible pathway of the Orbach process. The numbers provided at each arrow are the mean absolute value for the corresponding matrix element of transition magnetic moment. The first excited state is significantly high in energy (~200 cm^{-1}) from the ground state and at low-temperature the thermal energy will not be enough to populate the excited state. On other hand, the presence of large transverse component and hyperfine interactions opens a viable path which facilitates the strong QTM and enables the relaxation via ground state KDs. The zig-zag yellow lines represent the QTM as most-probable path for magnetic relaxation, which is in line with experimental observations.

$$\langle i | \mu | j \rangle = \frac{abs(\langle i | \mu_x | j \rangle) + abs(\langle i | \mu_y | j \rangle + abs(\langle i | \mu_z | j \rangle}{3} \qquad (3)$$
